# Production of itaconic acid from alkali pretreated lignin by dynamic two stage bioconversion

**DOI:** 10.1038/s41467-021-22556-8

**Published:** 2021-04-15

**Authors:** Joshua R. Elmore, Gara N. Dexter, Davinia Salvachúa, Jessica Martinez-Baird, E. Anne Hatmaker, Jay D. Huenemann, Dawn M. Klingeman, George L. Peabody, Darren J. Peterson, Christine Singer, Gregg T. Beckham, Adam M. Guss

**Affiliations:** 1 Biosciences Division, Oak Ridge National Laboratory, Oak Ridge, TN USA; 2 Biological Sciences Division, Pacific Northwest National Laboratory, Richland, WA USA; 3 National Bioenergy Center, National Renewable Energy Laboratory, Golden, CO USA; 4 Bredesen Center for Interdisciplinary Research, University of Tennessee, Knoxville, TN USA

**Keywords:** Metabolic engineering, Applied microbiology, Bacterial synthetic biology

## Abstract

Expanding the portfolio of products that can be made from lignin will be critical to enabling a viable bio-based economy. Here, we engineer *Pseudomonas putida* for high-yield production of the tricarboxylic acid cycle-derived building block chemical, itaconic acid, from model aromatic compounds and aromatics derived from lignin. We develop a nitrogen starvation-detecting biosensor for dynamic two-stage bioproduction in which itaconic acid is produced during a non-growth associated production phase. Through the use of two distinct itaconic acid production pathways, the tuning of TCA cycle gene expression, deletion of competing pathways, and dynamic regulation, we achieve an overall maximum yield of 56% (mol/mol) and titer of 1.3 g/L from *p*-coumarate, and 1.4 g/L titer from monomeric aromatic compounds produced from alkali-treated lignin. This work illustrates a proof-of-principle that using dynamic metabolic control to reroute carbon after it enters central metabolism enables production of valuable chemicals from lignin at high yields by relieving the burden of constitutively expressing toxic heterologous pathways.

## Abstract (web summary)

Lignin conversion to higher value products is essential to the economic viability of lignocellulosic biorefineries. Here, the authors demonstrate the bioconversion of alkali pretreated lignin to itaconic acid by dynamic two stage fermentation using a signal-amplified nitrogen-limitation biosensor.

## Introduction

**Fig. 1:**
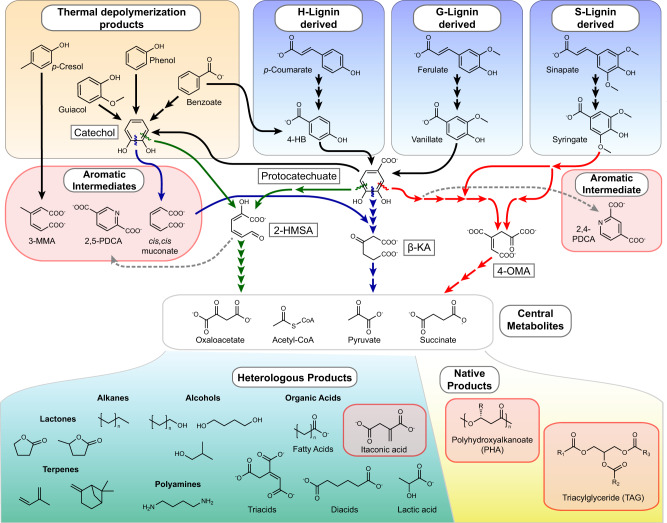
Biological upgrading of lignin by funneling depolymerized lignin aromatics toward value added products up and downstream of central metabolism. Solid colored and black arrows indicate known metabolic pathway steps for conversion of aromatic intermediates into central metabolites, with dotted black arrows indicating predicted metabolic pathway steps. Dotted gray lines indicate heterologous pathways to convert aromatic intermediates to value-added aromatic derivatives. Red boxed compounds are those whose production from deconstructed lignin have been demonstrated, including itaconate from this study. Acronyms used above: 4-HB (4-hydroxybenzoate), 3-MMA (3-methylmuconate), 2,5-PDCA (2,5-pyridinedicarboxylate), 2,4-PDCA (2,4-pyridinedicarboxylate), β-KA (β-ketoadipate), 2-HMSA (2-hydroxymuconate semialdehyde), 4-OMA (4-oxalomesaconate).

Valorization of lignin, a complex aromatic heteropolymer and the second most abundant component of terrestrial biomass, will be critical for the economic viability of lignocellulosic biorefineries^1^. Biological upgrading of lignin has been demonstrated with production of aromatic catabolic intermediates^2–7^ and their derivatives^8^, as well as carbon storage compounds such as polyhydroxyalkanoates (PHAs)^9,10^ and lipids^11^ (Fig. 1, red boxes). However, the sizes of individual chemical markets are typically at least an order of magnitude smaller when compared to fuel markets. With an estimated billion tons of plant biomass able to be sustainably grown in the United States for lignocellulosic biofuel production^12^, hundreds of millions of tons of lignin-rich feedstock could be available for valorization in the United States alone. Therefore, lignin will need to be converted into a wide array of products to avoid oversaturating individual chemical markets and to replace petroleum-derived incumbent molecules.

To increase the portfolio of products that can be made from lignin, additional parts of metabolism will need to be targeted. The tricarboxylic acid (TCA) cycle is a potential source of valuable chemicals including succinate and citrate, but it has not yet been harnessed for lignin valorization. Indeed, TCA cycle-derived chemicals are ideal products for lignin valorization because aromatic carbon is typically funneled directly into this part of metabolism. Itaconic acid is an unsaturated dicarboxylic acid derived from cis-aconitate in the TCA cycle, with industrial uses including as an acrylate alternative and for the production of polymers.^13^ Itaconic acid has been produced industrially from simple sugars, primarily glucose, since the 1950s^14,15^, and its potential to functionally replace several petroleum-derived commodity chemicals was highlighted by its selection as one of the top bio-based platform chemicals in several reports, including a 2004 United States Department of Energy report.^16^ However, the relatively high cost of sugars makes itaconic acid production expensive, limiting it to use as a specialty chemical. Using cheap and abundant feedstocks, such as lignin, has the potential to reduce production costs^17^ and enable much broader industrial use of itaconic acid.

**Fig. 2:**
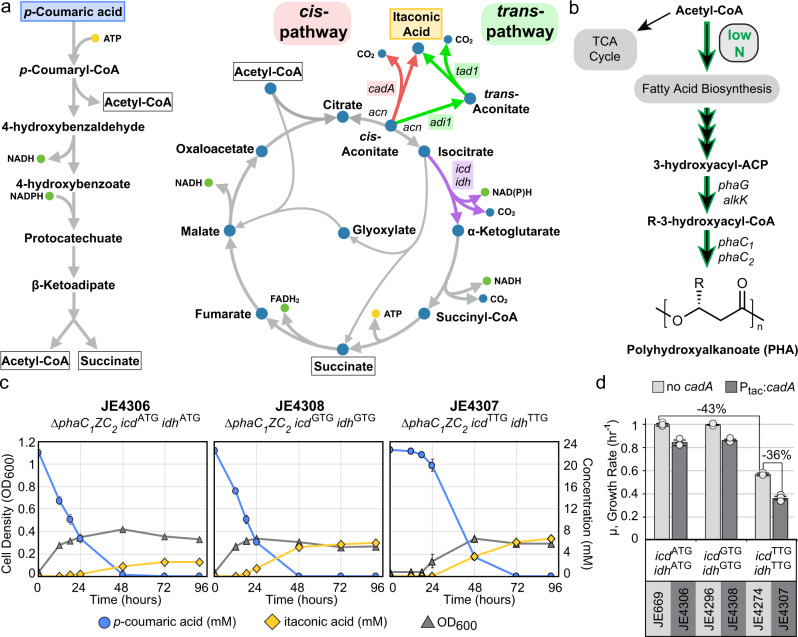
Two-stage production of itaconic acid from *p*-coumaric acid. **a** Simplified *p*-coumaric acid assimilation, β-ketoadipate, and tricarboxylic acid (TCA) cycle pathways in *Pseudomonas putida* KT2440 with modified or heterologous steps indicated by colored arrows, and connecting metabolites outlined. For simplicity some steps are not included. The cis (red arrow) and trans (green arrow) pathways for itaconic acid are indicated with involved genes, *cadA* (*cis*) & *tad1/adi1* (*trans*) adjacent to the reaction their gene products perform. Isocitrate dehydrogenase activity, provided by the *icd* & *idh* gene products, is indicated by a purple arrow. **b** Simplified polyhydroxyalkanoate (PHA) biosynthesis pathway in *P. putida* KT2440. The PHA pathway, via fatty acid biosynthesis, competes with the TCA cycle for acetyl-CoA during nitrogen-limited conditions. **c** Production of itaconic acid from *p-*coumaric acid in shake flasks by *P. putida* strains constitutively expressing *cadA* under nitrogen-limited conditions. Strain name and their unique modifications are indicated above the charts. Cell density (OD600, gray diamonds), residual *p*-coumaric acid (mM, blue circles), and produced itaconic acid (mM, yellow triangles) are indicated. **d** Growth rates of *P. putida* strains containing *icd* & *idh* start codon mutations with or without constitutive *cadA* expression using *p*-coumaric acid as sole carbon source. Rates were determined by 48-well microtiter plate cultivation. **c,** **d** Data are represented as mean values ± standard deviation in three replicates. Source data underlying Fig. 2c and d is provided as a Source Data file.

The saprophytic bacterium *Pseudomonas putida* KT2440 is a microbe of industrial interest^18,19^ due to its robust metabolism^20^ and tolerance to xenobiotics.^21–24^ *P. putida* also has the ability to tolerate and catabolize a wide-range of aromatic compounds^25^ which led to its recent use in upgrading depolymerized lignin into PHAs^10,26^, *cis,cis-*muconic acid^3,5,27^, and other intermediates in aromatic catabolism^7^. In *P. putida*, *p-*hydroxyphenyl (H) and guiacyl (G) lignin-derived aromatics are funneled via the β-ketoadipate pathway to acetyl-CoA and succinate (Fig. 2a). This direct route to key TCA cycle intermediates suggests that high yields of TCA cycle-derived products such as itaconic acid should be possible from lignin. For instance, because the lignin-derived aromatic compound *p*-coumaric acid is catabolized into one succinate and two acetyl-CoA molecules, the theoretical maximum yield of itaconic acid is 1.33 mol itaconic acid/mol *p-*coumaric acid.

Growth phase production of itaconic acid may be challenging because itaconic acid disrupts bacterial growth via inhibition of enzymes in the glyoxylate shunt^28^ and citramalate cycle^29^. An alternate approach is to use a two-stage process to decouple growth of the microbial catalyst from conversion of feedstocks to chemicals, which provides solutions to many problems present in growth-associated processes (e.g., product toxicity, slow catalyst growth).^30^ Such processes often take advantage of the natural responses to nutrient limitations (e.g., nitrogen, sulfur, phosphate) and environmental shifts (e.g., O_2_ limitation, temperature shifts) that prevent microbial growth while maintaining the metabolic reactions of interest. Coupling two-stage processes with dynamic metabolic control has the potential to entirely remodel metabolism.

In this study, we engineer *P. putida* to produce a commercially relevant chemical at high yields and gram-per-liter titers from model aromatic substrates and corn stover-derived, alkali-pretreated lignin. We further develop multiple production pathways and developed a signal-amplified biosensor for two-stage production via dynamic metabolic control to increase efficiency and mitigate toxicity.

## Results

### Initial production of itaconic acid from aromatic compounds

The enzyme *cis-*aconitate decarboxylase, encoded by the *Aspergillus terreus cadA* gene^31^, produces itaconic acid by enzymatic decarboxylation of the TCA cycle intermediate *cis*-aconitate (Fig. 2a). Using a previously developed site-specific DNA integration system in *P. putida* KT2440^32^, we integrated a constitutively expressed, codon optimized copy of *cadA* (P_tac_:*cadA*) into the genome of *P. putida* strain JE90 (named JE4305), and measured itaconic acid production from *p-*coumaric acid, the most abundant aromatic monomer released from corn stover during alkaline treatment^3^, as the sole carbon source. Under nitrogen-replete conditions, we were unable to detect itaconic acid production.

*P. putida* diverts carbon toward fatty acid biosynthesis for PHA production during nitrogen-starvation conditions (Fig. 2b).^9,10^ Thus, we hypothesized that more carbon would be directed to itaconic acid production under similar conditions. As predicted, nitrogen-starvation conditions enabled detectable itaconic acid production, but the yield was low (4.2% mol/mol) (Fig. 2c; Table 1). Because PHA production competes for carbon, we deleted the PHA synthetases *phaC*_*1*_ and *phaC*_*2*_ (strain named JE4306), increasing overall itaconic acid yield by approximately 3-fold (12% mol/mol) (Fig. 2c; Table 1). However, the majority of itaconic acid was produced during stationary phase, supporting the notion that more carbon is directed to itaconic acid production during nitrogen-starvation. During the non-growing stationary phase from 24 to 96 h, also referred to as the production phase, the itaconic acid yield from the remaining *p-*coumaric acid increased from undetectable production during growth phase to 33% mol itaconic acid /mol *p-*coumaric acid.

**Table 1:** Production of itaconic acid from *p*-coumaric acid during nitrogen limitation.

Strain	Hosted production pathway	Relevant parent genotype	Overall molar yield (mol/mol)	Stationary phase molar yield* (mol/mol)	Mass yield (g/g)	Titer (g/L)
JE4305	P_tac_:*cadA* (*cis*)	JE90 (*Pseudomonas putida* KT2440 *∆hsdR::Bxb1int-attB*)	0.04	0.10	0.03	0.12
JE4306	P_tac_:*cadA* (*cis*)	JE90 *∆phaC*_*1*_*ZC*_*2*_	0.12	0.33	0.09	0.12
JE4308	P_tac_:*cadA* (*cis*)	JE90 *∆phaC*_*1*_*ZC*_*2*_ *icd*^*GTG*^*:idh*^*GTG*^	0.27	0.72	0.21	0.78
JE4307	P_tac_:*cadA* (*cis*)	JE90 *∆phaC*_*1*_*ZC*_*2*_ *icd*^*TTG*^*:idh*^*TTG*^	0.30	0.97**	0.24	0.90
JE3221	P_T7_:*cadA* (*cis*)	JE90 P_urtA_:*T7pol*:P_cat_:*lysY ∆phaC*_*1*_*ZC*_*2*_	0.09	0.18	0.07	0.22
JE3713	P_T7_:*cadA* (*cis*)	JE90 P_urtA_:*T7pol*:P_cat_:*lysY ∆phaC*_*1*_*ZC*_*2*_ *icd*^*GTG*^*:idh*^*GTG*^	0.29	0.79	0.23	0.81
JE3715	P_T7_:*tad1:adi1* (*trans*)	JE90 P_urtA_:*T7pol*:P_cat_:*lysY ∆phaC*_*1*_*ZC*_*2*_ *icd*^*GTG*^*:idh*^*GTG*^	0.43	1.02	0.34	1.09
JE3717	P_T7_:*cadA* (*cis*)	JE90 P_urtA_:*T7pol*:P_cat_:*lysY ∆phaC*_*1*_*ZC*_*2*_ *icd*^*TTG*^*:idh*^*TTG*^	0.51	0.97	0.40	1.27
JE3719	P_T7_:*tad1:adi1* (*trans*)	JE90 P_urtA_:*T7pol*:P_cat_:*lysY ∆phaC*_*1*_*ZC*_*2*_ *icd*^*TTG*^*:idh*^*TTG*^	0.56	1.16	0.45	1.26^#^

*Stationary phase molar yield was calculated using itaconic acid yield from 24 to 96 h time points.

**Stationary phase molar yield was calculated using 48 to 96 h time points and may be not directly comparable with other samples.

#Some residual *p*-coumaric acid remained unutilized after 96 h.

All data are represented as the mean of three replicates.

### Modulating TCA cycle flux increases itaconic acid yields and titers

As an obligate aerobe, *P. putida* maintains robust TCA cycle activity for energy production. We hypothesized that reducing flux through isocitrate dehydrogenase (Fig. 2a – *icd, idh*) should increase accumulation of *cis*-aconitate, and therefore increase yields. However, deletion of *icd* and *idh* would make *P. putida* an energy-starved, α-ketoglutarate auxotroph, and likely unable to grow on lignin-derived substrates. Thus, we aimed to reduce the translation efficiency of *icd* and *idh*, which are encoded next to each other on the chromosome and divergently transcribed. Using reporter gene fusions, we showed that changing the translational start codon of the fluorescent protein mNeonGreen to GTG or TTG reduced fluorescence per cell by 2.3- and 4.2-fold, respectively (Supplementary Table 1), suggesting that this approach could be used to decrease the level of TCA cycle enzymes. Therefore, we altered the start codons of *icd* and *idh* such that they were each GTG or TTG, generating strains JE4296 (*icd*^GTG^:*idh*^GTG^) and JE4274 (*icd*^TTG^:*idh*^TTG^), respectively. Cell yield (as measured by final OD_600_) was largely unaffected by the start codon alterations (Supplementary Fig. 1a). The growth rate of JE4296 was also unaffected, while the growth rate of JE4274 on *p-*coumaric acid was decreased by 43% (Fig. 2d, Supplementary Fig. 1a).

To determine the impact of these mutations on itaconic acid production, we integrated the P_tac_:*cadA* cassette into both strains, generating strains JE4308 (*icd*^GTG^:*idh*^GTG^, P_*tac*_*:cadA*) and JE4307 (*icd*^TTG^:*idh*^TTG^, P_*tac*_*:cadA*), and assayed itaconic acid production from *p*-coumaric acid under nitrogen-limited and nitrogen-replete conditions. Slowing the TCA cycle was sufficient to allow detectable itaconic acid production under nitrogen-replete conditions (Supplementary Fig. 1b), and nitrogen-limited conditions increased overall yields from 12 to 27% and 30% mol/mol with JE4308 and JE4307, respectively (Fig. 2c; Table 1). During the non-growing production phase, itaconic acid yield increased to 72% mol/mol for strain JE4308 and 97% for strain JE4307. While yields improved, the detrimental effect of constitutive *cadA* expression was highlighted by decreased growth rates in strains expressing *cadA* (Fig. 2d). Growth rates in all three genetic backgrounds were negatively impacted by constitutive *cadA* expression, suggesting that expression of *cadA* may be toxic. The impact of *cadA* expression was most pronounced in JE4307, where it caused a 36% reduction in growth rate over parent strain JE4274.

### Development of a signal-amplified nitrogen-limitation biosensor for dynamic metabolic control in *P. putida* KT2440

**Fig. 3:**
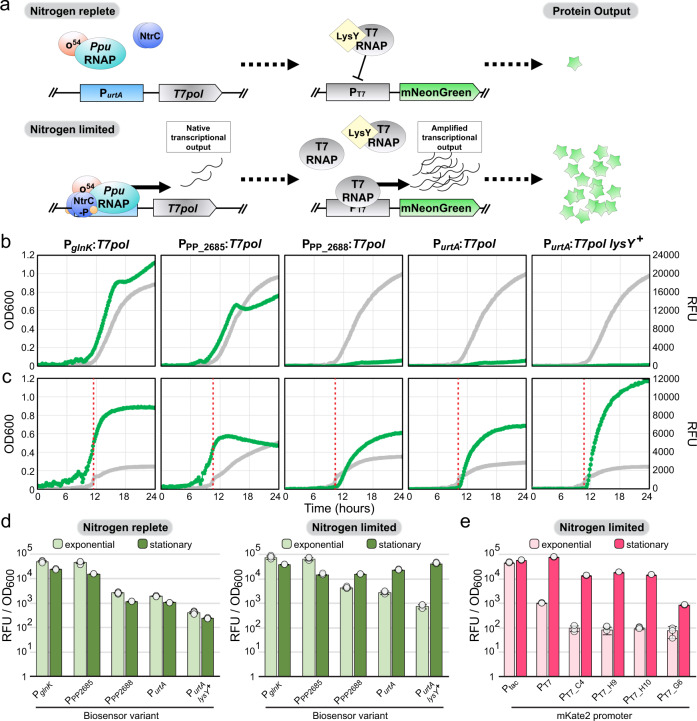
Development of a nitrogen-limitation biosensor to enable dynamic control of two-stage bioproductions. **a** Diagram of biosensor design and utilization as a regulated signal amplifier for pathway and tool expression. *P. putida* RNA polymerase (Ppu RNAP), sigma factor 54 (σ54), T7 RNA polymerase gene (T7pol), wavy line (mRNA), T7 RNA polymerase protein (T7 RNAP), green stars (mNeonGreen protein). Representative growth curves of triplicate cultures for 96-well microtiter plate cultivations of candidate biosensor strains, biosensor variant indicated, with integrated P_*T7*_:mNeonGreen cassette in either nitrogen-replete (**b**) or nitrogen-limited (**c**) medium. Cell density and mNeonGreen production, as measured by OD600 (gray) and relative fluorescence units (RFU—green) respectively, were measured every 10 min. Entry to stationary phase is indicated (red dotted line) for nitrogen-limited cultures. **d** Graph of mNeonGreen production by candidate biosensor sensor strains during exponential growth (light green) or stationary phase (dark green) in microtiter plate cultivations. **e** Graph of mKate2 production by JE2113-derivatives with integrated P_*T7*_-variant:mKate2 cassettes during exponential growth (light pink) or stationary phase (dark pink) in plate reader cultivations. **d, e** Data are presented as the mean values ± standard deviation in three replicates. Source data underlying Fig. 3b–e is provided as a Source Data file.

Limiting the expression of the apparently toxic CadA protein to the production phase via dynamic regulation could substantially improve both growth and itaconic acid production. Therefore, we pursued development of an expression system that is induced upon nitrogen starvation. Furthermore, native regulatory systems are typically tuned to provide expression sufficient for associated pathways, which is often insufficient for heterologous pathways. However, use of an orthogonal RNA polymerase intermediary such as the T7 RNA polymerase (T7pol)^33^ can allow amplification of the original signal^34^ (Fig. 3a).

In the absence of preferred nitrogen sources, such as ammonium, *P. putida* activates genes for both the nitrogen-starvation response and utilization of alternative nitrogen-sources^35,36^. To identify a nitrogen starvation-sensitive promoter in *P. putida*, we performed transcriptomics during growth with a preferred (ammonia) or alternative (nitrate) nitrogen source (Table 2 and Supplementary Table 2). Based on the combination of these results and previous studies^35,36^, we selected four candidate promoters to test as biosensors: P_PP_2685_, P_PP_2688_, P_*urtA*_, and P_*glnK*_. Candidate promoters were used to express T7pol and integrated into the JE90 genome along with the gene encoding fluorescent protein mNeonGreen under control of a T7 promoter. Strains were grown under nitrogen-replete and nitrogen-limited conditions (Fig. 3b–d). While the P_*glnK*_ and P_PP_2685_ promoters were surprisingly nitrogen-agnostic, displaying constitutive mNeonGreen expression similar to the σ^70^ *tac* promoter (Supplementary Fig. 2), the other candidates P_PP_2688_ and P_*urtA*_ responded to nitrogen-limitation, demonstrating 3.7 and 8.8-fold mNeonGreen induction upon entry into nitrogen-depletion-induced stationary phase (Fig. 3d).

**Table 2:** Differential expression of genes downstream of potential nitrogen-sensitive promoters in *P. putida* JE1657.

Locus Tag	Gene Name	log_2_ fold change (NaNO_3_ / NH_4_Cl)	Base mean	Predicted gene function
PP_1705	*nirB*	8.14	2029.14	nitrite reductase large subunit
PP_2092	*nasA*	6.31	361.67	nitrate transporter
PP_2094	*nasS*	2.79	51.23	nitrate binding protein
PP_2685	*—*	4.44	320.51	bacterial proteasome, beta subunit
PP_2688	*—*	3.99	132.41	circularly permuted ATP-grasp type 2
PP_2842	*ureD*	4.38	181.83	urease accessory protein
PP_4053	*treY*	2.19	1151.28	maltooligosyl trehalose synthase
PP_4841	*urtA*	4.37	455.68	urea ABC transporter substrate-binding protein
PP_4842	*urtB*	4.56	72.42	urea ABC transporter permease
PP_4845	*urtE*	3.77	67.21	ABC transporter ATP-binding protein
PP_5234	*glnK*	1.45	8496.51	NRII(GlnL/NtrB) phosphatase activator

Values represent the output of DEseq2 software package using four replicates for each nitrogen source.

While the initial P_*urtA*_ biosensor variant allowed strong induced expression, basal expression in the presence of nitrogen was relatively high. To reduce basal T7pol activity, we constitutively expressed a catalytically-deactivated variant of T7 lysozyme (LysY)^37^, which allosterically inhibits T7pol activity^38^ (Fig. 3a). The expression of LysY substantially improved biosensor performance, decreasing basal mNeonGreen expression by 78% in exponential phase and increasing the maximal induced mNeonGreen expression level. This resulted in a 60-fold mNeonGreen induction, a 6.8-fold improvement over the strain lacking LysY (Fig. 3d). As an orthogonal measurement of biosensor performance, we utilized RNAseq to compare *T7pol* gene expression in strain JE2212 (P_*urtA*_:*T7pol*:*lysY* + P_T7_:*mNeonGreen*) with ammonium or nitrate as the sole nitrogen source as done above. Highlighting the function of this biosensor as a signal amplifier, NO_3_-induced *mNeonGreen* mRNA abundance was 302- and 54-fold higher than *urtA* and *T7pol*, respectively (Supplementary Data 1).

Optimal pathway performance often requires tuning expression of individual proteins. Tuning expression is often achieved with promoter^32,39^ and/or RBS^40^ modifications. We utilized a small library of T7 promoter variants^39^ with the red fluorescent protein mKate2 to tune the magnitude of biosensor outputs. Unlike the σ^70^ *tac* promoter (Supplementary Fig. 2a) which was constitutively expressed, nitrogen-limitation was required for induction of mKate2 production in all five T7 promoter variants (Fig. 3e, Supplementary Table 3). Within the promoter library, maximum protein expression levels varied over an 89-fold range. Interestingly, we observed a 2-3.5-fold dynamic range improvement over the standard T7 promoter with three of the variant promoters—largely driven by considerably lower basal expression—which approached the background autofluorescence.

### Dynamic regulation improves two-stage production of itaconic acid production from lignin-derived aromatics

**Fig. 4:**
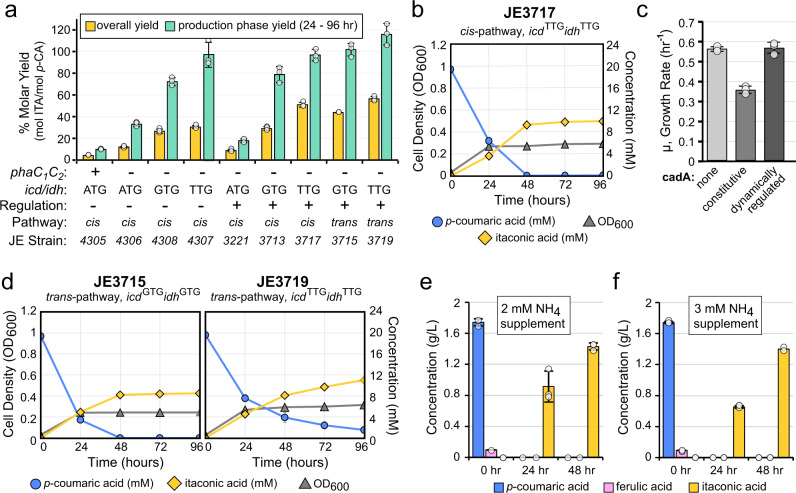
Biosensor-controlled expression of itaconic acid production pathways enables high yield production from lignin and model aromatic substrates. **a** Molar yield of engineered strains from shake flask experiments with 20 mM *p-*coumaric acid as sole carbon source. Overall yield (yellow) and production phase yield (green) are indicated. Production phase was defined as 24 hr to 96 hr time points. Relevant genetic modifications include the presence (+) or deletion (−) of the native PHA polymerase genes *phaC*_*1*_*C*_*2*_, the start codon used (ATG, GTG, or TTG) for isocitrate dehydrogenase genes *icd* and *idh*, use of a constitutive (−) or dynamically regulated (+) itaconic acid biosynthesis pathway, the *cis*- or *trans*- itaconic acid production pathway, and the corresponding stain name. (**b,d**) Production of itaconic acid from *p-*coumaric acid in shake flasks by strains utilizing dynamically-regulated (**b**) *cadA* (cis-pathway) or (**d**) *tad1*/*adi1* (trans-pathway) under nitrogen-limited conditions. Strain names and their unique modifications are indicated above the charts. Cell density (OD600, gray diamonds), *p-*coumaric acid (mM, blue circles), and itaconic acid (mM, yellow triangles) are indicated. **c** The effect of *cadA* expression on growth of *P. putida icd*^TTG^ *idh*^TTG^ strains in 48-well microtiter plate assays with *p-*coumaric acid as sole carbon source. Consumption of detected aromatic monomers and production of itaconic acid from depolymerized lignin containing either (**e**) 2 mM or (**f**) 3 mM supplemented NH_4_Cl in shake flask cultivations with strain JE3715. **a**−**f** Data are represented as the mean ± standard deviation in three replicates, with the exception of 48 h time point in panel **f**, which only contains two replicates and has no error bars. Source data are provided as a Source Data file.

We next tested whether dynamic regulation of *cadA* would improve itaconic acid production by combining the altered isocitrate dehydrogenase start codons and the P_urtA_:*T7pol*:*lysY*^*+*^ biosensor cassette. The original T7 promoter showed the highest maximal expression level, so we integrated *cadA* under the control of this T7 promoter into all three strains (named JE3221, JE3713, and 3717; Table 1), and assayed production of itaconic acid from *p*-coumaric acid under nitrogen-limited conditions. Similar to previous shake flask experiments—with the exception of JE4307 (P_*tac*_:*cadA*, *icd*^TTG^:*idh*^TTG^)—growth is complete within the first 24 h, with some itaconic acid production occurring, likely after growth is completed. Itaconic acid yields with JE3221 (P_T7_:*cadA*, *icd*^ATG^:*idh*^ATG^) and JE3713 (P_T7_:*cadA*, *icd*^GTG^:*idh*^GTG^) were similar to their corresponding constitutive *cadA* strains (Figs. 2c, 4a). Strains JE3713 (P_T7_:*cadA*) and JE4308 (P_tac_:*cadA*) also displayed similar yield from remaining *p-*coumaric acid in the non-growing production phase, 72 and 79% mol/mol, respectively. Strain JE3717 (P_T7_:*cadA*, *icd*^TTG^:*idh*^TTG^), on the other hand, achieved an overall itaconic acid yield of 51% mol/mol (Fig. 4a, Table 1), a 67% improvement over the best performing constitutive *cadA* expression strain (Fig. 2c). Furthermore, dynamic regulation of *cadA* eliminated the growth defect induced by *cadA* expression (Fig. 4c), which has ramifications on itaconic acid productivity—at 48 h JE3717 (P_T7_:*cadA*, *icd*^TTG^:*idh*^TTG^) itaconic acid production is essentially complete (Fig. 4b), while production by JE4307 (P_tac_:*cadA*, *icd*^TTG^:*idh*^TTG^) was not complete after 72 h (Fig. 2c). Taken together, dynamic regulation of *cadA* improves performance, and it will likely improve strain stability by eliminating the growth defect.

### Metabolic pathway selection to optimize itaconic acid production

To date, other than in some organisms that natively produce itaconic acid, attempts to engineer strains for itaconic acid production have focused on the heterologous expression of the cis-aconitate decarboxylase (termed here the *cis-*pathway) from *A. terreus*. However, an alternate pathway for itaconic acid production was recently discovered in *Ustilago maydis*.^41^ This pathway, referred to here as the trans-pathway, proceeds through two steps. First, *cis*-aconitate is isomerized by aconitate isomerase (*adi1*) to the thermodynamically favorable isomer *trans-*aconitate, which is subsequently decarboxylated by *trans*-aconitate decarboxylase (*tad1*) to generate itaconic acid (Fig. 2a). The *trans* isomer comprises 88% of aconitate at equilibrium and is a competitive inhibitor of the aconitase enzyme^42^—both features that could increase substrate accumulation and therefore increase flux to itaconic acid.

Taken together, we hypothesized that the *trans*-pathway would improve itaconic acid production relative to the *cis-*pathway by providing a thermodynamically favorable route to divert carbon flux from the TCA cycle. To test this, we integrated codon-optimized *tad1* and *adi1* genes under the control of the T7 promoter into strains JE3674 (*icd*^GTG^:*idh*^GTG^) and JE3681 (*icd*^TTG^:*idh*^TTG^) and assayed the resulting strains (JE3715 and JE3719, respectively) for itaconic acid production (Fig. 4d). As hypothesized, strains expressing the *trans*-pathway from *U. maydis* produced higher molar yields than equivalent strains expressing the cis-pathway (Fig. 4c, Table 1), and JE3719 produced the highest itaconic acid yield (56% mol/mol overall yield) from *p-*coumaric acid in this study, with a production phase yield of 116% mol/mol which is 88% of the maximum theoretical yield of 1.33 mol itaconic acid/mol *p-*coumarate.

### Production of itaconic acid from depolymerized lignin and other substrates

To test the viability of itaconic acid production from lignin, we assayed the ability of strain JE3715 to upgrade a depolymerized lignin stream produced from a lignocellulose deconstruction process^3^ to itaconic acid. Base-catalyzed depolymerization of corn stover lignin was performed as described previously^3^, and the resulting liquor was diluted with concentrated modified M9 salts containing either 2 or 3 mM NH_4_Cl. The liquor provides 10.7 mM (1.74 g/L) *p*-coumaric acid, 0.5 mM (0.09 g/L) ferulic acid, trace amounts of other monomeric carbon sources, and residual higher molecular weight lignin. JE3715, which contains the dynamically regulated *trans-*pathway and the GTG start codons for *icd* and *idh*, was chosen as the biocatalyst as its substantially higher growth rate and relatively similar yield enables much higher volumetric productivity relative to JE3717, and because JE3719 did not completely consume the provided substrate (Table 1). This strain was inoculated into shake flasks containing the two media variants and assayed for itaconic acid production. Production of itaconic acid leveled off at 48 h with titers between 1.40 and 1.43 g/L (Fig. 4e–f). The high apparent yields (99% overall molar yield and 0.79 g itaconic acid/g aromatic monomer) suggest that there may be other components in the lignin stream that lead to product and/or that the strain is breaking down and utilizing oligomeric lignin, as previously described^43^. Finally, to demonstrate the general utility of this organism and pathway, we demonstrated itaconic acid production from other potential waste stream feedstocks, including other aromatic compounds (benzoic acid, ferulic acid), sugars (glucose, xylose, arabinose), glycerol, and organic acids (acetic acid, succinic acid, octanoic acid) (Supplementary Table 4). With JE3715, the overall itaconic acid yield from ferulic acid (51.6% mol/mol) was similar to *p-*coumaric acid in the same experiment (56% mol/mol), and overall yield from benzoic acid reached 24% of the theoretical maximum yield without any optimization.

## Discussion

Until recently, robust itaconic acid production was limited to sugar-utilizing fungi such as *A. terreus* and *U. maydis*, but in recent years, bacterial strains have been engineered to produce itaconic acid from glucose^44,45^, acetate^46^, and glycerol.^47^ However, all efforts to engineer heterologous itaconic acid production relied on expression of the *cis*-pathway (*cadA*) from *A. terreus*.^31^ In our work, the *trans*-pathway (*adi1/tad1*) from *U. maydis*^41^ outperformed the *cis*-pathway, likely due to the production of a thermodynamically favorable intermediate. Accordingly, the *trans*-pathway may improve performance in other organisms. Among bacteria, the itaconic acid yield with *P. putida*, 0.44 g/g from *p-*coumaric acid, compares well to an engineered *E. coli* that produced 0.5 g itaconic acid/g glucose.^45^ Furthermore, 1.43 g/L itaconic acid was produced from a complex, depolymerized lignin stream, yielding 0.79 g itaconic acid/g detectable aromatic monomers. Of note, the yield during the production phase is substantially higher than the overall yield in all cases, reaching nearly 1.2 mol itaconic acid/mol *p-*coumaric acid (the theoretical maximum yield is 1.33 mol/mol) using trans*-*pathway strains (Fig. 4c, Table 2). Therefore, advanced feeding strategies with extended production phases could improve yields even further. Finally, this work demonstrates itaconic acid production in an engineered bacterium in minimal salts medium without the use of replicating plasmids, antibiotic selections, or expensive inducer chemicals (*e.g*. IPTG), each of which will be critical for a commercial process.

Dynamic metabolic control combined with two-stage production is promising approach for biological chemical production^30^, and the biosensor developed here can serve as a master regulator for additional dynamic metabolic control tools such as CRISPRi^48^, targeted proteolysis, and nested dynamic regulatory systems. Our nitrogen biosensor will be a valuable tool for future *P. putida* metabolic engineering because it is able to tune the amplitude of the nitrogen-starvation induced transcriptional response over an 89-fold range, increasing it by up to 60-fold over the original response. Despite its utility, to the best of our knowledge, *lysY* has not previously been used for metabolic engineering. The dynamic response of this biosensor, already allowing 200-fold induction, has the potential to be further tuned by altering *lysY* expression. Although not explicitly demonstrated here, one can modulate levels of sequentially utilized nitrogen sources such as NH_4_ and NO_3_ for auto-induction of gene expression in single-stage processes following depletion of NH_4_. Finally, nitrogen-responsive σ^54^ promoters from *P. putida* similar to P_*urtA*_ have been ported to *E. coli*^36^, suggesting this biosensor can work in other organisms expressing the activator NtrC.

In addition to lignin, recent work has demonstrated the potential for *P. putida* to valorize other biomass streams – including thermochemical wastewater^49^ and lignocellulosic sugar hydrolysates^50^. Here, we demonstrated itaconic acid production from multiple substrates that could be found in potential commercial feedstocks, including biodiesel waste (glycerol), plant biomass (sugars, aromatics, acetic acid), and lipids (octanoic acid). Therefore, the technology developed here can serve as a universal platform for the two-phase production of a portfolio of chemicals from alternate carbon sources beyond lignin.

## Methods

### General culture conditions and media

The strains and plasmids used in this study are listed in Supplementary Table 5. Routine cultivation of *Escherichia coli* for plasmid construction and maintenance was performed at 37 °C using LB (Miller) medium supplemented with 50 µg/mL kanamycin sulfate and 15 g/L agar (for solid medium). All *Pseudomonas putida* cultures were incubated at 30 °C, with shaking at 250 rpm for shake flask cultures with *p*-coumarate, shaking at 225 rpm for BCDL medium shake flask cultures, and 548 rpm with a 2 mM orbital for cultures performed in a Neo2SM plate reader (BioTek). LB (Miller) was used for routine *Pseudomonas putida* strain maintenance, competent cell preparations, and starter cultures. For itaconate production assay starter cultures, the media was supplemented with 50 µg/mL kanamycin sulfate.

Modified M9 medium (M9*) with variable amounts of NH_4_Cl was utilized for shake flask experiments, growth rate assays, and fluorescent reporter assays (47.8 mM Na_2_HPO_4_, 22 mM KH_2_PO_4_, 8.6 mM NaCl, 1 mM MgCl_2_, 0.1 mM CaCl_2_, 18 µM FeSO_4_, 1x MME trace minerals, pH adjusted to 7 with KOH). In total, 1000x MME trace mineral stock solution contains per liter, 1 mL concentrated HCl, 0.5 g Na_4_EDTA, 2 g FeCl_3_, 0.05 g each H_3_BO_3_, ZnCl_2_, CuCl_2_·2H_2_O, MnCl_2_·4H_2_O, (NH_4_)_2_MoO_4_, CoCl_2_·6H_2_O, NiCl_2_·6H_2_O. Unless otherwise noted, all M9* medium was supplemented with 20 mM *p-*coumarate (neutralized with NaOH) as a sole carbon source. MME medium (containing 9.1 mM K_2_HPO_4_, 20 mM MOPS, 4.3 mM NaCl, 0.41 mM MgSO_4_, 68 µM CaCl_2_, 1x MME trace minerals, pH adjusted to 7.0 with KOH) supplemented with 20 mM glucose and either 20 mM NH_4_Cl or 20 mM NaNO_3_ was utilized for transcriptomics experiments.

### Production of BCDL and depolymerized lignin media preparation

The preparation of BCDL has been reported before.^32^ Specifically, dry solid material remaining from the enzymatic hydrolysis of pretreated corn stover (which follows the biorefinery process designed at NREL^51^) was added as 10% (w/v) solids to a 2% NaOH solution and loaded into 200 mL stainless steel reactors. The reaction was carried out at 120 °C for 30 min. The sterile and solubilized material was neutralized with 4 N H_2_SO_4_ and centrifuged at 10,875 × *g* for 20 min in aseptic conditions. Then, the supernatant (90% v/v) was mixed with 10x M9* salts (without any nitrogen source) and NH_4_Cl to generate M9*-BCDL medium supplemented with either 2 mM or 3 mM NH_4_Cl.

### Plasmid and *Pseudomonas* strain construction

Phusion® HF Polymerase (Thermo Scientific) and primers synthesized by Eurofins Genomics were used in all PCR amplifications for plasmid construction. OneTaq® (New England Biolabs - NEB) was used for colony PCR. Plasmids were constructed by Gibson Assembly using NEBuilder® HiFi DNA Assembly Master Mix (NEB) or ligation using T4 DNA ligase (NEB). Plasmids were transformed into either competent NEB 5-alpha F’I^q^ (NEB), Epi400 (Lucigen), or QP15 (Epi400 mated with NEB 5-alpha F’I^q^ to transfer the mini F’ plasmid to Epi400). Standard chemically competent *Escherichia coli* transformation protocols were used to construct plasmid host strains. Transformants were selected on LB (Miller) agar plates containing 50 µg/mL kanamycin sulfate for selection and incubated at 37 °C. Template DNA was either synthesized by IDT or isolated from *E. coli* or *P. putida* KT2440 using Zymo Quick gDNA miniprep kit (Zymo Research). Zymoclean Gel DNA recovery kit (Zymo Research) was used for all DNA gel purifications. Plasmid DNA was purified from *E. coli* using GeneJet plasmid miniprep kit (ThermoScientific) or ZymoPURE plasmid midiprep kit (Zymo Research). Sequences of all plasmids were confirmed using Sanger sequencing performed by Eurofins Genomics. Plasmids used in this work are listed in Supplementary Table 5, and details regarding plasmid construction and sequences are below. All DNA oligos used in this work can be found in Supplementary Table 6.

*P. putida* JE90, a derivative of *P. putida* KT2440 where the restriction endonuclease *hsdR* has been replaced with the Bxb1-phage integrase and respective *attB* sequence^32^, was used as a parent for all *P. putida* strains used in this study (Supplementary Table 5). All genome modifications were performed using either the homologous recombination-based pK18mobsacB kanamycin resistance/sucrose sensitivity selection/counter-selection system^52,53^ or with the Bxb1-phage integrase system^32^ with minor modifications to competent cell preparation procedures. Electrocompetent *Pseudomonas putida* were prepared as follows. Strains were inoculated into LB broth and incubated at 30 °C, 250 rpm until they reached stationary phase—typically ~16 h. Cells were centrifuged at 3000 × *g* for 20 min at room temperature and following decanting of supernatant they were washed by resuspended in 1/2 the original culture’s volume of room temperature 10% glycerol. Resuspended cells were centrifuged, decanted and resuspended in 10% glycerol two additional times. Following the final centrifugation, cells were resuspended in 1/50^th^ the original culture’s volume of 10% glycerol. Cells were either used immediately or stored at −80 °C. For transformation, 5 µL (200 ng-2 µg) of plasmid DNA was added to 50 µL of the electrocompetent cells, transferred to a 0.1 cm electroporation cuvette, and and electroporated at 1.6 kV, 25 uF, 200 Ω. Electroporated cells were resuspended following addition of 950 µL SOC medium, transferred to a 1.5 mL microcentrifuge tube, and incubated with 250 rpm shaking for 1 h at 30 °C. Dilutions of the recovery cultures were plated onto LB agar medium supplemented with 50 µg/mL kanamycin sulfate. For plasmids integrated using Bxb1 integrase transformants were screened by colony PCR to verify plasmid insertion into the genome. For strains constructed using homologous recombination, transformants were streaked for single colony isolation on LB agar medium supplemented with 50 µg/mL kanamycin sulfate and incubated overnight at 30 °C to select against residual wild-type cells that are insensitive to sucrose. For sucrose counter-selection, restreaked transformants were streaked for single colonies on YT + 25% sucrose plates (10 g/L yeast extract, 20 g/L tryptone, 250 g/L sucrose, and 18 g/L agar), and incubated at 30 °C overnight. Colonies were streaked a second time on YT + 25% sucrose, and screened by colony PCR for desired mutations. Gene deletions and replacements were performed by homologous recombination, while integration of reporter and itaconate production pathway cassettes was performed with the Bxb1-phage integrase system. Primers used for screening *P. putida* strains for *phaC*_*1*_*ZC*_*2*_ deletion, *ampC*::T7_RNAP replacements, and *icd/idh* start codon swaps can be found in Supplementary Table 6. Integration of pJE990-derivatives using the phage integrase system was confirmed by colony PCR using oligos oJE66 & oJE536.

### Plasmid construction details

Annotated sequences of all plasmids can be found in Supplementary Data 3. All enzymes used for plasmid construction were purchased from NEB. Supplementary Table 6 contains the sequence of all oligos listed below.

For construction of pJE473, homology arms to target deletion of *phaC*_*1*_*ZC*_*2*_ (PP_5003-5005) were amplified by PCR from wild-type *P. putida* genomic DNA using primer combinations oJE331/332 and oJE333/334, assembled into gel purified EcoRI/HindIII-linearized pJE382, and transformed into NEB 5-alpha F’I^Q^. Resulting *E. coli* colonies were screened by colony PCR for the presence of homology arms using primers oJE255/256. Candidates for pJE473 were purified from *E. coli* and sequenced using primers oJE255/256.

For construction of pJE387, homology arms to target deletion of *ampC* (PP_2876) were amplified by PCR from wild-type *P. putida* genomic DNA using primer combinations oJE89/90 and oJE91/92, assembled into gel purified EcoRI/HindIII-linearized pK18mobsacB, and transformed into NEB 5-alpha F’I^Q^. Resulting *E. coli* colonies were screened by colony for the presence of homology arms using primers oJE255/256. Candidates for pJE387 were purified from *E. coli* and sequenced using primers oJE255/256.

For construction of pJE382, we utilized QuikChange PCR mutagenesis with oligos oJE69/70 to amplify a version of pK18mobsacB with the lac promoter that drives expression of the lacZalpha fragment deleted. Following amplification, the PCR reaction was digested with 20 U of DpnI (NEB) for 1 h at 37 °C to reduce parental plasmid background. The purpose of this plasmid was to reduce likelihood of recombination with lac/tac-based promoters in pK18mobsacB with similar promoters in our synthetic constructs, and to reduce potential toxicity from unintended transcription of homology arms/expression cassettes in *E. coli*. The DpnI-digested PCR product was transformed into NEB 5-alpha F’I^Q^. Resulting *E. coli* colonies were screened by colony for the presence of the lac promoter using primers oJE71/72. Candidates for pJE382 were purified from *E. coli* and lac promoter deletion verified by sequencing with primer oJE72.

For construction of pJE1031, homology arms for the deletion of *ampC* (PP_2876) were amplified from pJE387 using primer combination oJE92/608, assembled into gel purified EcoRI/HindIII-linearized pJE382, and transformed into NEB 5-alpha F’I^Q^. Resulting *E. coli* colonies were screened by colony PCR for the presence of homology arms using primers oJE255/256. Candidates for pJE1031 were purified from *E. coli* and sequenced using primers oJE255/256.

For construction of pJE1032, pJE1033, pJE1037, and pJE1039 promoter sequences containing ~200-300 bp upstream of PP_2685, PP_2688, *urtA* (PP_4841), and *glnK* (PP_5234), respectively, were amplified from *P. putida* (Supplementary Table 6 for oligos) and assembled with T7 RNAP and a synthetic terminator sequence. The T7 RNAP polymerase and a downstream terminator was amplified from BL21(DE3) *pLysS* (Promega) genomic DNA using oligos oJE625/626. A double terminator sequence for insulation of the construct was amplified from the T7_dbl_term gBlock using oJE627/628. Parts were assembled into BamHI/XbaI-linearized pJE1031, and transformed into NEB 5-alpha F’I^Q^. Resulting *E. coli* colonies were screened by colony PCR using primers oJE177/178. Candidates for the plasmids were purified from *E. coli* and sequenced using oJE177/178/631/632/633.

For construction of the reporter plasmids we annealed oligos containing desired promoter sequences and ligated the promoters into a promoterless mNeonGreen reporter plasmid, pJE990. Plasmid pJE990 was linearized with BbsI. Promoter oligos pairs were phosphorylated with PNK (NEB) in T4 DNA ligase buffer, annealed by heating to 95 °C and cooling at 1 °C/minute to room temperature. Annealed oligo sets oJE634/635, oJE97/98/133/134, oJE826/827, oJE828/829, oJE830/831, and oJE832/833 were ligated to BbsI-linearized pJE990 to construct plasmids pJE1040, pJE1045, pJE1118, pJE1119, pJE1120, and pJE1121 respectively. Ligated DNA was transformed into NEB 5-alpha F’I^Q^. Plasmids were isolated from transformant colonies and confirmed by sequencing with oJE535. For construction of mKate2 variant plasmids, mKate2 was amplified from the mKate2 gBlock using oligos oJE1724/1725 and digested with NdeI/XbaI. Plasmids pJE1045, pJE1040 and pJE1118-1121 were digested with NdeI/XbaI and ligated with NdeI/XbaI digested mKate2 gBlock to generate plasmids pGW55, pJE1454-1458. Ligations were transformed into NEB 5-alpha F’I^Q^, and candidates confirmed by sequencing of isolated plasmid DNA using oligos oJE535/536.

For construction of pJE1180 we amplified the *cat* and *lysS* genes from pLysS (Promega) as two parts with primers designed to introduce the *lysY* mutation, assembled the resulting parts into SpeI-linearized pJE1040. Primers oJE817/818 and oJE819/820 were used to amplify the two parts. The resulting *lysY/cat* fragment was digested with SpeI and ligated into XbaI-linearized pJE1037, generating plasmid pJE1180.

For construction of pJE1380, codon-optimized *cadA* from *Aspergillus terreus* was assembled into NdeI/XbaI-linearized pJE1040—replacing mNeonGreen. The *cadA* gene was synthesized as gBlocks cadA_gBlock_1 & cadA_gBlock_2. gBlocks 1 & 2 were amplified using oligos oJE1408/1409 and oJE1410/1411, respectively. The assembly was transformed into NEB 5-alpha F’I^Q^, and transformants were screened using oJE535/536. Plasmid DNA was isolated from PCR positive candidates and sequenced using oJE535/536/1412.

For pJE1390, the *cadA* gene from pJE1380 was excised using NdeI/XbaI, and ligated into NdeI/XbaI linearized pJE1045. The ligation was transformed into QP15, and transformants were screened by colony PCR using oligos oJE535/536. The assembly was transformed into NEB 5-alpha F’I^Q^, and transformants were screened using oJE535/536. Plasmid DNA was isolated from PCR positive candidates and sequenced using oJE535/536/1412.

For pJE1443, codon-optimized *tad1* and *adi1* genes from *Ustilago maydis* were assembled into AflIII/XbaI-linearized pJE1040 - replacing mNeonGreen and its RBS sequence. The *tad1* and *adi1* genes were synthesized as gBlocks tad1 and adi1, which were amplified using primer combinations oJE1554/1547 and oJE1555/1548, respectively. The assembly was transformed into NEB 5-alpha F’I^Q^, and transformants were screened using oJE535/536. Plasmid DNA was isolated from PCR positive candidates and sequenced using oJE535/536/1559/1560/1561.

For the construction of the *icd* / *idh* start codon swap plasmids pJE1444 and pJE1445, we assembled several PCR reactions containing homology arms for targeting, and mutations in the start codons (and RBS neutral mutations in the region between core RBS and start codon) of *icd* & *idh*. The homology arms for targeting insertion of the two plasmids into the *icd/idh* locus were amplified using primer pairs oJE1564/1565 and oJE1568/1569 for both plasmids. The central fragment contained between the two homology arms, containing the various mutations, was amplified using oligos oJE1566/1567 for pJE1444 and oligos oJE1570/1571 for pJE1445. The parts were assembled into EcoRI/HindIII-linearized pJE382, transformed into NEB 5-alpha F’I^Q^, and transformants were screened using oJE255/256. Plasmid DNA was isolated from PCR positive candidates and sequenced using oJE255/256/1572/1573.

For construction of pJE365, homology arms to target deletion of *gcd* (PP_1444) were amplified by PCR from wild-type *P. putida* genomic DNA, assembled into gel purified XbaI/HindIII-linearized pK18mobsacB, and transformed into NEB 5-alpha F’I^Q^. Resulting *E. coli* colonies were screened by colony for the presence of homology arms using primers oJE255/256. Candidates for pJE365 were purified from *E. coli* and sequenced using primers oJE255/256.

For construction of plasmid pJE1345, we had IDT synthesize gBlocks containing a *P. putida* codon-optimized *araA*_*2*_*-araE*_*2*_ oxidative L-arabinose catabolic pathway cassette from *Burkholderia ambifaria* AMMD and a *P. putida* codon-optimized *E. coli araE*_*1*_ L-arabinose:H^+^ symporter cassette. The gBlocks were assembled in between ∆*gcd* homology arms in pJE365 backbone and transformed into NEB 5-alpha F’I^Q^. Resulting *E. coli* colonies were screened by restriction digest for the presence of the properly assembled *B. ambifaria araCDABE* cassette. Candidates for pJE1345 were purified from *E. coli* and confirmed by Sanger sequencing. See plasmid map for sequence details.

For construction of pJE1479, we had IDT or GenScript synthesize portions of a cassette that contains a *P. putida* codon-optimized *E. coli xylE* xylose:H^+^ symporter expression cassette that is divergent from a *P. putida* codon-optimized *Burkholderia xenovorans xylCDBX* oxidative xylose catabolic pathway expression cassette. These cassettes were assembled using Gibson Assembly with homology arms to target insertion downstream of *fpvA* in the *Pseudomonas putida* KT2440 genome. See plasmid map for sequence details.

Plasmid pJE1045 was PCR amplified using primers pJE1045-ATGGTCf / pJE1045-ATGGTCr, pJE1045-GTGGTCf / pJE1045-GTGGTCr, and pJE1045-TTGGTCf / pJE1045-TTGGTCr to produce plasmids pJE1045-ATGGTC, pJE1045-GTGGTC, and pJE1045-ATGGTC, respectively by PCR mutagenesis. The resulting PCR products were digested with DpnI, transformed into NEB 5-alpha F’I^Q^. Plasmids from resulting colonies were screened by Sanger sequencing.

### Growth rate analysis

LB medium was inoculated from glycerol stocks and incubated overnight at 30 °C, 250 rpm for precultures. Cultures were washed twice by centrifugation (~4000 × *g* for 10 min) and resuspension in equal volumes of 1x M9 salts lacking NH_4_Cl to remove residual LB medium and resuspended in 1/3 volume 1x M9 salts. Optical density (OD_600_) of resulting suspensions was measured using a 1 cm pathlength cuvette. Growth assays were performed with 600 uL M9* medium supplemented with 20 mM *p-*coumarate and 20 mM NH_4_Cl in clear 48-well microtiter plates with an optically clear lid (Greiner Bio-One). All cultures were inoculated with washed cultures to an OD_600_ equivalent to 0.03 in a 1 cm pathlength cuvette. Plates were incubated at 30 °C, fast shaking in an Epoch2 plate reader (Bio-Tek) using Gen5 software (V3.0), with OD_600_ readings taken every 10 minutes. Exponential growth rates were determined using the CurveFitter software (Version 1; http://www.evolvedmicrobe.com/CurveFitter/) with data points in early mid-log phase. All growth rates were calculated from 3 replicate experiments. Standard deviations are two-sided.

### Fluorescent reporter assays

Strains were revived from glycerol stocks in 5 mL LB with overnight incubation at 30 °C, 250 rpm. 5 mL starter cultures in M9* + 20 mM glucose + 10 mM NH_4_Cl were inoculated with 1% of the recovery culture and similarly incubated. Coupled growth and fluorescence assays were performed with a Neo2SM (Bio-Tek) plate reader using 200 µL/well of M9* + 20 mM *p-*coumarate + 2 mM (limiting) or 20 (replete) mM NH_4_Cl in black-walled, µClear® flat-bottom, 96-well plates (Greiner Bio-One) with an optically clear lid. Plate cultures were inoculated with 0.5% inoculum from starter cultures, and incubated overnight at 30 °C, fast shaking with OD_600_ and fluorescence (F_510,530_ for mNeonGreen and F_588,633_ for mKate2) measured every 10 minutes. Reporter expression per cell was estimated by dividing relative fluorescence units (RFU) by OD_600_ (as a proxy for cell number) for each time point and averaging those values for time points occurring during either exponential growth or stationary phase. Background absorbance and fluorescence readings from wells containing media blanks were averaged and subtracted from sample readings prior to analysis. Exponential phase was defined as time points where OD_600_ was between 0.039 and the OD_600_ curve inflection point, typically OD_600_ ~0.2 (nitrogen limited) or ~0.6 (nitrogen replete). Stationary phase was defined as time points starting 2 h following end of exponential phase. Standard deviations are two-sided.

### Shake flask experiments for itaconate production

Starter cultures were prepared as described for growth rate assays with the exception that 50 µg/mL kanamycin sulfate was added to the medium. Starter cultures were inoculated to a final OD_600_ of 0.1 (*p-*coumarate cultures) or 0.2 (BCDL cultures) into 25 mL of M9*-coumarate or M9*-BCDL medium, supplemented with either 2 mM NH_4_Cl (all *p-*coumarate cultures) or 3 mM (BCDL cultures), in a 125 mL Erlenmeyer flask and incubated at 30 °C, 250 rpm. Cultures were sampled periodically to measure growth by OD_600_, and analyte concentrations by high performance liquid chromatography (HPLC).

### Analytical techniques

For shake flask experiments with M9*-*p*-coumarate, optical density at 600 nm (OD_600_) was measured directly from cultures using a spectrophotometer (Amersham, UltroSpec10) blanked with M9*-coumarate. HPLC analysis for *p-*coumarate and organic acid detection was performed by injecting 20 µL of 0.2 µm filtered culture supernatant onto a Waters 1515 series system equipped with a Rezex RFQ-Fast Acid H + (8%) column (Phenomenex) and a Micro-Guard Cation H^+^ cartridge (Bio-Rad). Samples were run with column at 60 °C using a mobile phase of 0.01 N sulfuric acid at a flow rate of 0.6 mL/min, with a refractive index detector and UV/Vis detector measuring A_230_ & A_280_ for analyte detection. Analytes were identified and quantified by comparing retention times and spectra with pure standards.

For shake flask experiments with M9*-BCDL, optical density at 600 nm (OD_600_) was measured with a Nanodrop (ThermoFisher Scientific) after diluting samples sixfold. Uninoculated M9*-BCDL medium was used as a blank to subtract signal coming from components in the medium.

Itaconic acid quantitation in M9*-BCDL. Prior the analysis, a 0.1 mL aliquot was taken from each sample and 0.9 mL of water were added to make a 10x dilution. Then, 34 µL of 72% sulfuric acid were added to each diluted sample to decrease the pH below 2.0 and precipitate acid insoluble lignin. Samples were centrifuged, and the supernatant was filtered through a 0.2 µM filter pore size. Itaconic acid quantification was performed on an Agilent 1100 series HPLC system, with a diode array detector (DAD) at 210 nm (Agilent Technologies). Analysis was performed by injecting 6 µL of filtered culture supernatant onto a Phenomenex Rezex™ RFQ-Fast Acid H^+^(8%) column with a cation H^+^guard cartridge (Bio-Rad Laboratories) at 85 °C using a mobile phase of 5 mM sulfuric acid at a flow rate of 1.0 mL/min.

Aromatic compounds quantitation in M9*-BCDL. Metabolite analysis in BCD was performed on an Agilent 1200 LC system (Agilent Technologies) equipped with a DAD. Each sample and standard was injected at a volume of 10 μL onto a Phenomenex Luna C18(2) column 5 μm, 4.6 × 150 mm column (Phenomenex). The column temperature was maintained at 30 °C and the buffers used to separate the analytes of interest were (A) 0.05% acetic acid in water and (B) 0.05% acetic acid in acetonitrile. The chromatographic separation was carried out using a gradient of: initially starting at 1% B going to 50% B at 35 min before immediately switching to 99% B at 35.1 min, before equilibrium for a total run time of 47 min. The flow rate of the mobile phases was held constant at 0.6 mL/min. The same standards used in the BCDL experiments were also used to construct calibration curves, but between the ranges of 5–200 µg/L. Three separate wavelengths from the DAD were used to identify and quantitate the analytes of interest. A wavelength of 210 nm and 225 nm was used for the analytes vanillic acid and 4-hydroxybenzoic acid. A wavelength of 325 nm was used for the analytes *p-*coumaric acid, and ferulic acid. A minimum of five calibration levels was used with an r^2^ coefficient of 0.995 or better for each analyte. Standard deviations are two-sided.

### Transcriptional profiling of *P. putida*

For the determination of NO_3_ induced promoters, we utilized strain JE1657, an engineered *P. putida* strain containing a Bxb1 phage integrase system for rapid genomic integration of DNA^32^, and a P_T7_:mNeonGreen reporter cassette. JE1657 was cultured at 30 C in 50 mL MME mineral medium in a 250 mL Erlenmeyer shake flask at 30 °C, 250 rpm shaking and harvested mid-log (OD_600_ = ~0.2) by centrifugation (~16,000 × *g*, 2 min, 4 °C). Supernatants were quickly decanted, and cell pellets were frozen rapidly in liquid nitrogen prior to storage at −80 °C for storage prior to RNA isolation. Four samples were prepared for each condition. For characterization of biosensor performance, strain JE2212 was cultured under identical conditions.

Cell pellets, were resuspended in TRIzol (ThermoFisher-Invitrogen, Waltham, MA USA) and processed according to the manufactures protocol for TRIzol reagent. In general, TRIzol was added to cell pellets and mixed by vortex and pipetting. Chloroform was then added and mixed and samples were centrifuged. After centrifugation the aqueous layer was removed and mixed 1:1 with 80% ethanol. The samples were then purified on a RNeasy column (Qiagen Hilden, Germany) following the manufactures protocol and the on-column DNase digestion. RNA was eluted off the column in 35 µL RNAse free H_2_0 (Qiagen, Hilden, Germany). RNA concentration was quantified using a Nanodrop 1000 instrument (ThermoScientific, Waltham, MA) and RNA quality was verified by obtaining RNA Integrity Numbers (RIN) using an RNA 6000 Nanochip on an Agilent 2100 Bioanalyzer (Agilent Technologies, Santa Clara, CA).

Ribosomal RNA was depleted from total RNA samples using a RiboZero rRNA Removal Kit (Epicentre-Illumina Inc. San Diego, CA) according to manufacturer’s instructions. The depleted sample was purified on a RNA Clean & Concentrator-5 (Zymo Research, Irvine, CA, USA) following the manufacturer’s protocol, and then the depleted material was quantified using a Nanodrop 1000 and visualized on an Agilent 2100 Bioanalyzer instrument with a RNA 6000 Nanochip (Agilent Technologies, Santa Clara, CA). RNA depleted of ribosomal RNA was used as input material to synthesize cDNA libraries using a ScriptSeq v2 RNA-Seq Library Preparation Kit (Illumina-Epicentre, San Diego, CA, USA) according to manufacturer’s instructions and TruSeq compatible barcodes. Pooled barcoded libraries were sequenced in one direction for 50 bases (SE50) on an Illumina Hi-Seq2500 using v4 chemistry (Illumina Inc. San Diego, CA) and de-multiplexed as a sequencing service provided by The Genomic Services Lab at Hudson Alpha Institute for Biotechnology (HudsonAlpha, Huntsville, AL).

### Differential gene expression analysis

After Illumina sequencing, RNA-seq reads were mapped to modified versions of the *P. putida* KT2440 reference genome (NC_002947) containing the mutations found in JE1657 and JE2212 using the Geneious for RNA-seq mapping workflow. Read count per annotated gene was calculated for each treatment and replicate, as well as fragment per kilobase million (FPKM), a common normalization technique. We then exported gene locus tags and raw read counts into tab-delimited files, one for each replicate. To calculate differential gene expression, we used the R package DESeq2^54^ (version 1.10.0), which calculates log-fold change in expression and allows comparison between treatments using several replicates. We had three (JE2212 assay) or four (JE1657 assay) replicates per treatment, for a total of six or eight inputs per experiment. Differential expression data can be found in Supplementary Data 1 (JE2212) and Supplementary Data 2 (JE1657).

### Statement on measurements

For all data points in this manuscript, measurements were taken from distinct samples.

### Reporting summary

Further information on research design is available in the Nature Research Reporting Summary linked to this article.

## Supplementary information

Supplementary Information file

Supplementary Data 1

Supplementary Data 2

Supplementary Data 3

Description of Additional Supplementary Files

Reporting Summary

## Data Availability

Data supporting the findings of this work are available within the paper and its Supplementary Information files. A reporting summary for this Article is available as a Supplementary Information file. The datasets, plasmids, and microorganisms generated and analyzed during the current study are available from the corresponding author upon request. NGS expression data generated in this study were deposited in the Gene Expression Omnibus (GEO) under accession number GSE147420. Source data are provided with this paper.

## Source data

Source Data
