# Supplementary material for: Production of itaconic acid from alkali pretreated lignin by dynamic two stage bioconversion
Source: Nat Commun. 2021 Apr 15;12:2261. doi: 10.1038/s41467-021-22556-8 (PMC8050072; doi:10.1038/s41467-021-22556-8)
Supplement: Supplementary file 1 — Supplementary Information file [file 41467_2021_22556_MOESM1_ESM.pdf]

Production of itaconic acid from alkali pretreated lignin by dynamic two stage bioconversion

Elmore *et al.*

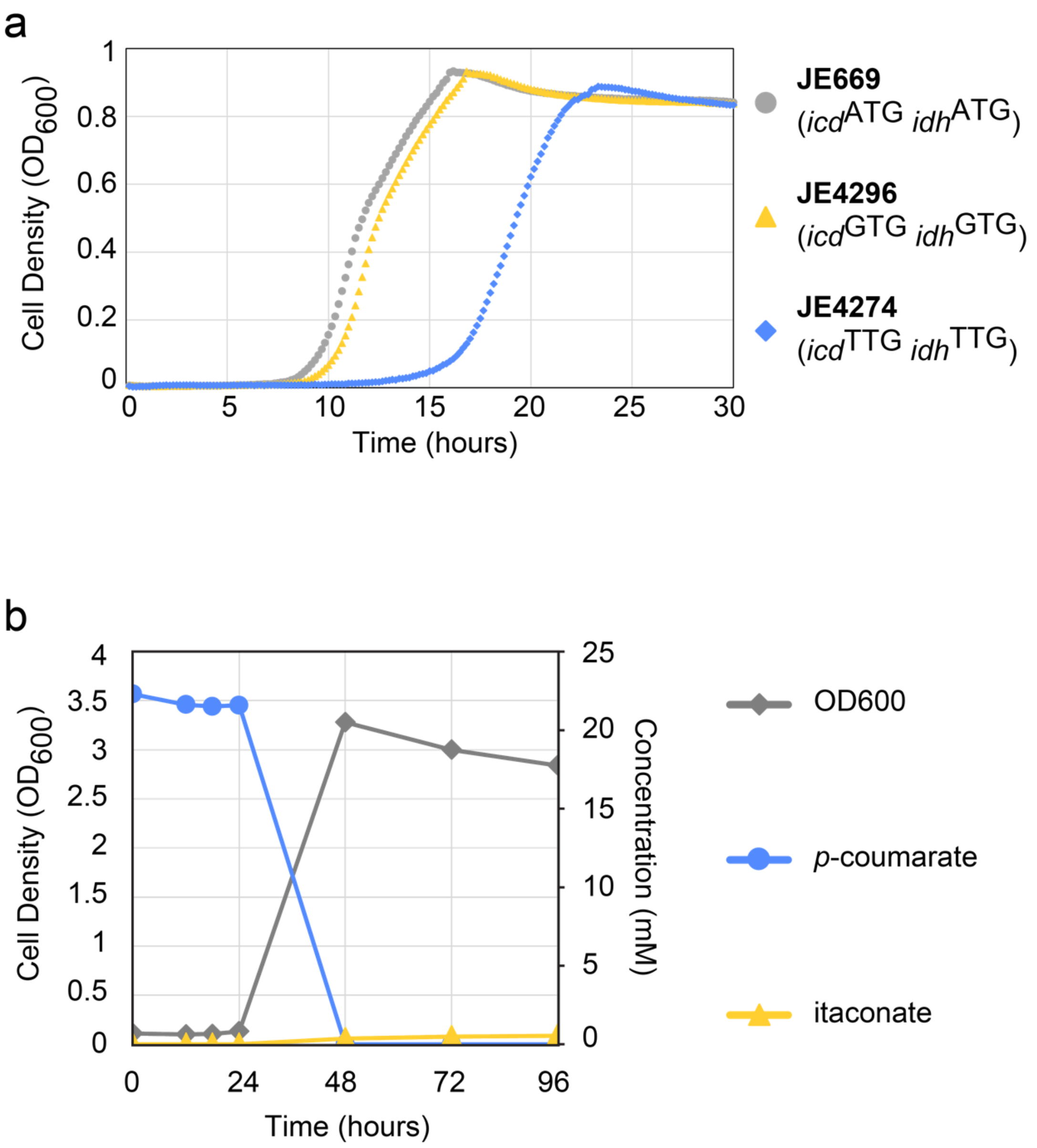

Supplementary Figure 1. Effect of reduced isocitrate dehydrogenase expression on growth and itaconate production by Pseudomonas putida KT2440. (a) Microtiter plate growth assay of *P. putida* strains harboring wild-type (gray circle), moderately reduced (yellow triangle), or strongly reduced (blue diamond) isocitrate dehydrogenase activity with *p*-coumarate as sole carbon source. Growth curves displayed are the mean of three biological replicates. (b) Two-stage production of itaconic acid from *p*-coumaric acid in the presence of excess nitrogen (20 mM NH_4) by engineered *P. putida* strain JE4307 (constitutive *cadA, icd*^{TTG} *idh*^{TTG}) in shake flasks. Cell density (OD600, gray diamonds), residual *p*-coumaric acid (mM, blue circles), and produced itaconic acid (mM, yellow triangles) are indicated. Data are represented as the mean +/- standard deviation in three replicates. Source data are provided as a Source Data file.

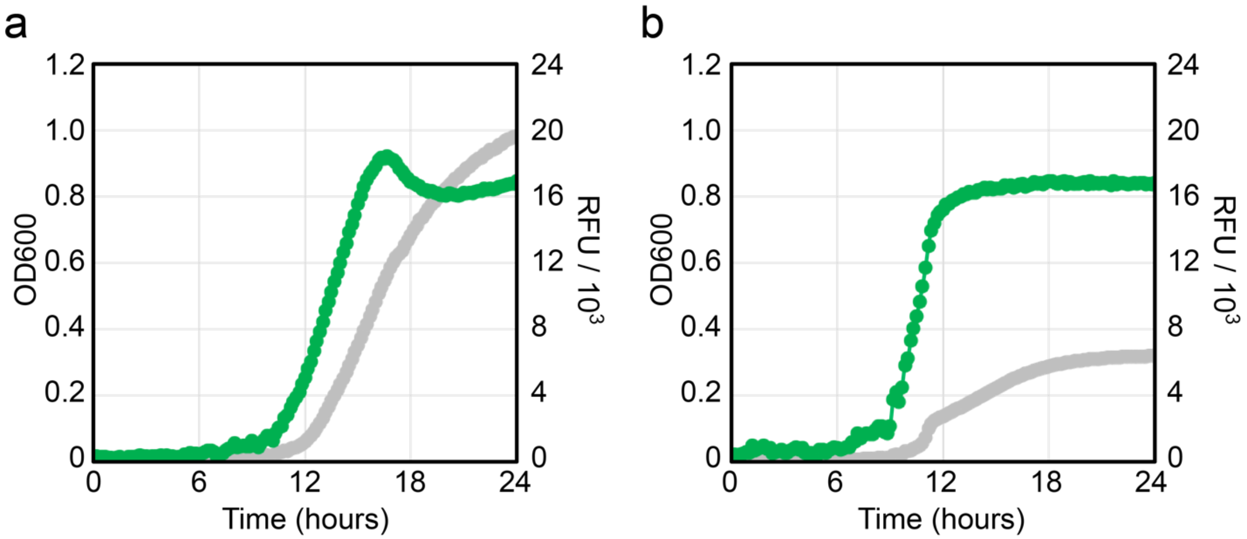

Supplementary Figure 2. mNeonGreen production by constitutive promoter (Ptac) in a nitrogen-biosensor strain. Representative growth curves of triplicate cultures for 96-well microtiter plate cultivations of candidate biosensor strain JE2113 (P_{urtA}:T7 RNAP, *lysY*$^{+}$) with integrated (constitutive) P_{tac} controlled mNeonGreen cassette. Strain was grown in either nitrogen-replete (a) or nitrogen-limited conditions (b). Cell density and mNeonGreen production, as measured by OD600 (gray) and relative fluorescence units (RFU - green) respectively, were measured every 10 minutes. All growth experiments were repeated in triplicate. Source data are provided as a Source Data file.

Supplementary Table 1. Reduction in mNeonGreen expression when using alternate start codons.

mNeonGreen Start Codon	Carbon source	Predicted translation initiation rate (TIR) for mNeonGreen*	Predicted fold-reduction in RFU/OD relative to ATG	Fluorescence / Cell (RFU/OD)	Observed fold-reduction in RFU/OD relative to ATG
ATG	20 mM glucose	846.56	---	8268 +/- 153	---
ATG	20 mM *p*-coumaric acid	846.56	---	3174 +/- 126	---
GTG	20 mM glucose	467.37	1.81	3615 +/- 127	2.29
GTG	20 mM *p*-coumaric acid	467.37	1.81	1416 +/- 27	2.24
TTG	20 mM glucose	212.63	3.98	1882 +/- 101	4.39
TTG	20 mM *p*-coumaric acid	212.63	3.98	801 +/- 29	3.96

Data are represented as the mean +/- standard deviation in three replicates. *TIR calculated using the DenovoDNA RBS calculator (www.denovodna.com). Source data are provided as a Source Data file.

Supplementary Table 2. Differential gene expression - NO3 vs. NH4 in JE1647.

Locus Tag in NC_002947	Gene	baseMean	log_2 fold change ($NaNO_3/NH_4Cl$)*	padj	First gene in operon?	Entire operon is differentially regulated >4-fold?	Function likely directly related to nitrogen source metabolism?
PP_0233	tauA CDS	259.98	2.49	0.095930536	yes	no	yes, taurine
PP_0806	surface adhesion protein CDS	1504.44	2.03	1.23E-23	yes	yes	no
PP_0808	hmp CDS	1919.02	4.59	0.010280754	yes	yes	no
PP_1703	assimilatory nitrate reductase/sulfite reductase CDS	474.75	4.72	3.77E-118	yes	yes	yes, nitrate/nitrite
PP_1705	nirB CDS	2029.14	8.14	2.94E-221	yes	yes	yes, nitrite
PP_1706	nirD CDS	1103.25	7.91	4.65E-160	no	yes	yes, nitrite
PP_1707	D-isomer specific 2-hydroxyacid dehydrogenase family protein CDS	85.81	3.03	2.92E-27	no	no	no
PP_2036	4-hydroxy-tetrahydrodipicolinate synthase CDS	86.41	2.24	3.18E-09	no	no	no
PP_2037	aldolase CDS	103.16	2.44	9.34E-13	yes	no	no
PP_2090	cobA CDS	436.93	6.20	2.81E-92	no	yes	unclear
PP_2091	serine/threonine-protein kinase CDS	287.70	5.48	1.01E-80	no	yes	yes, serine/threonine
PP_2092	nasA CDS	361.67	6.31	5.47E-64	yes	yes	yes, nitrate
PP_2093	two-component system response regulator NasT CDS	17.90	3.82	6.30E-13	no	yes	yes
PP_2094	nitrate-binding protein NasS CDS	51.23	2.79	2.59E-11	yes	yes	yes, nitrate
PP_2106	ammonium transporter CDS	219.11	2.63	1.03E-11	yes	yes	yes, ammonium
PP_2685	hypothetical protein CDS	320.51	4.44	3.96E-69	yes	yes	unclear
PP_2686	transglutaminase domain-containing protein CDS	84.59	4.77	2.17E-46	no	yes	yes, glutamine
PP_2687	hypothetical protein CDS	106.97	3.99	7.40E-41	no	yes	yes
PP_2688	hypothetical protein CDS	132.41	3.99	2.75E-18	yes	yes	unclear
PP_2762	sfnA CDS	9725.44	10.39	NA	yes	yes	no
PP_2842	ureD CDS	181.83	4.38	1.01E-23	yes	yes	yes, urea
PP_2843	ureA CDS	53.56	4.36	2.05E-22	no	yes	yes, urea
PP_2844	ureB CDS	123.15	4.63	2.58E-39	no	yes	yes, urea
PP_2845	ureC CDS	394.55	4.47	2.16E-76	no	yes	yes, urea
PP_2846	ureE CDS	110.23	5.35	1.93E-42	no	yes	yes, urea
PP_2847	ureJ CDS	85.40	4.86	2.23E-61	no	yes	yes, urea
PP_2848	ureF CDS	241.52	4.87	2.31E-40	no	yes	yes, urea
PP_2849	ureG CDS	357.18	4.87	1.03E-78	no	yes	yes, urea
PP_3375	endA CDS	117.21	2.21	9.54E-07	no	yes	no
PP_3376	ptxD CDS	606.57	3.19	8.33E-09	no	yes	no
PP_3377	kguT CDS	978.47	2.71	6.43E-06	no	yes	no
PP_3378	kguK CDS	646.22	2.93	1.25E-07	no	yes	no
PP_3379	kguE CDS	424.53	2.50	4.49E-05	yes	yes	no
PP_4053	treY CDS	1151.28	2.19	2.23E-15	no	no	no, glycogen
PP_4604	DMT superfamily permease CDS	546.30	3.49	4.11E-30	yes	no	yes, branched chain amino acids
PP_4605	AraC family transcriptional regulator CDS	125.05	2.03	7.35E-20	no	no	yes, branched chain amino acids
PP_4841	urtA CDS	455.68	4.37	1.40E-52	yes	yes	yes, urea
PP_4842	urtB CDS	72.42	4.56	1.93E-42	yes	yes	yes, urea
PP_4843	urtC CDS	74.54	4.17	4.64E-33	no	yes	yes, urea
PP_4844	urtD CDS	99.89	5.26	2.04E-57	no	yes	yes, urea
PP_4845	urtE CDS	67.21	3.77	6.91E-34	yes	yes	yes, urea
PP_5233	amtB CDS	1082.62	5.46	2.35E-76	yes	yes	yes, ammonium
PP_5234	glnK CDS	8496.51	1.45	0.002360948	yes	yes	yes, regulator of nitrogen metabolism

p-values (pvalue) are calculated using the likelihood ratio test in DESeq2 to compare treatments (NO3 and NH4) for each gene individually in a single test. FDR/Benjamini-Hochberg adjusted *p*-values (padj) are corrected for multiple testing, with values of NA indicating a potential false positive.

[*]Positive log2foldchange = higher expression in NO3 versus NH4.

Supplementary Table 3. T7 promoter variant testing.

Promoter	T7 promoter variant sequence	mKate2 (RFU/OD600) in exponential	mKate2 (RFU/OD600) in stationary	Fold-induction in N-limited stationary phase
P_{tac} (constitutive)	---	43755 ± 1546	54287 ± 572	1.24 ± 0.03
P_{T7}	taatacgactcactaTAGGGgaa	979 ± 30	73036 ± 2563	74.67 ± 4.76
P_{T7_C4}	taatacgactcactaTCAAGgaa	95 ± 24	12847 ± 416	142.61 ± 36.75
P_{T7_H10}	taatacgactcactaCGGAAgaa	79 ± 28	17782 ± 301	262.27 ± 13.88
P_{T7_H9}	taatacgactcactaATACTgaa	91 ± 9	14110 ± 126	157.16 ± 17
P_{T7_G6}	taatacgactcactaTTTCGgaa	74 ± 38	817 ± 12	15.92 ± 12.22

Data are represented as the mean +/- standard deviation in three replicates. Source data are provided as a Source Data file.

Supplementary Table 4. Itaconic acid production from other potential waste stream feedstocks.

Strain	Carbon source	Nitrogen source	Maximum theoretical yield (mol ITA/mol substrate)	Itaconic acid at 48 hours (mM)	Yield at 48 hours (mol ITA/mol substrate)	% of theoretical yield at 48 hours	Itaconic acid at 72 hours (mM)	Yield at 72 hours (mol ITA/mol substrate)	% of theoretical yield at 72 hours
JE3903*	20 mM Glucose	2 mM NH4Cl	1	7.76 +/- 3.88	0.388	38.80%	8.27 +/- 1.03	0.414	41.35%
	20 mM Xylose	2 mM NH4Cl	0.5	2.51 +/- 1.25	0.126	25.10%	2.93 +/- 0.28	0.147	29.30%
	20 mM Arabinose	2 mM NH4Cl	0.5	3.78 +/- 1.98	0.189	37.80%	4.09 +/- 0.57	0.205	40.90%
JE3715	20 mM *p*-coumaric acid	2 mM NH4Cl	1.33	9.67 +/- 3.97	0.484	36.35%	11.31 +/- 0.3	0.566	42.52%
	20 mM ferulic acid	2 mM NH4Cl	1.33	6.99 +/- 2.87	0.350	26.28%	10.32 +/- 1.34	0.516	38.80%
	20 mM benzoic acid	2 mM NH4Cl	1	3.82 +/- 1.61	0.191	19.10%	4.8 +/- 1.59	0.240	24.00%
	30 mM acetic acid	2 mM NH4Cl	0.33	0.52 +/- 0.29	0.017	5.25%	0.59 +/- 0.16	0.020	5.96%
	15 mM octanoic acid	2 mM NH4Cl	1.33	1.85 +/- 1.09	0.123	9.27%	2.36 +/- 0.47	0.157	11.83%
	30 mM succinic acid	2 mM NH4Cl	0.5	1.46 +/- 0.79	0.049	9.73%	1.58 +/- 0.54	0.053	10.53%
	40 mM glycerol	2 mM NH4Cl	0.5	3.18 +/- 1.35	0.080	15.90%	4.35 +/- 0.95	0.109	21.75%

*JE3903 is a derivative of JE3681 (P_{urtA}:*T7pol*:*lysY*+ *icd*^{TTG},*idh*^{TTG}) that has been engineered for catabolism of pentoses that native *P. putida* is unable to catabolize. The heterologous pathways incorporated enable catabolism of xylose and arabinose via oxidative pathways that enter central carbon metabolism at alpha-ketoglutarate, limiting the maximum itaconic acid yield to 0.5 mol itaconic acid/mol pentose sugar. The strain utilizes *cadA* for itaconic acid production. See Supplementary Table 5 for strain genotype. These data represent the mean and standard deviation from four replicates. Source data are provided as a Source Data file.

Supplementary Table 5. Strains and plasmids used in this work.

Name	Relevant genotype	Source
Strains		
NEB 5-alpha F'Iq	*Escherichia coli* F´ *proA*$^{+}$*B*$^{+}$ *lacI*^{q} *Δ(lacZ)M15 zzf::Tn10* (Tet^{R}) */ fhuA2Δ(argF-lacZ)U169 phoA glnV44 Φ80Δ(lacZ)M15 gyrA96 recA1 relA1 endA1 thi-1 hsdR17*	New England Biolabs
Epi400	*Escherichia coli* F^{-} *mcrA Δ(mrr-hsdRMS-mcrBC) Φ80dlacZΔM15 ΔlacX74 recA1 endA1 araD139 Δ(ara, leu)7697 galU galK λ*$^{-}$ *rpsL (Str*^{R}*) nupG trfA tonA pcnB4 dhfr*	Lucigen
QP15	*Escherichia coli* F´ *proA*$^{+}$*B*$^{+}$ *lacI*^{q} *Δ(lacZ)M15 zzf::Tn10* (Tet^{R}) */ mcrA Δ(mrr-hsdRMS-mcrBC) Φ80dlacZΔM15 ΔlacX74 recA1 endA1 araD139 Δ(ara, leu)7697 galU galK λ*$^{-}$ *rpsL (Str*^{R}*) nupG trfA tonA pcnB4 dhfr*	this work
BL21 (DE3) pLysS	*Escherichia coli* F- , *omp*T, *hsd*S$_{B}$ (r$_{B}$–, m$_{B}$–), *dcm*, *gal*, λ(DE3), pLysS, Cm^{r}.	Promega
JE90	*Pseudomonas putida* KT2440 *ΔhsdR::Bxb1int-attB*	[1]
JE1622	*P. putida KT2440 ΔhsdR::Bxb1int-attB ΔampC::*P_{PP2685}*:T7pol*	this work
JE1626	*P. putida KT2440 ΔhsdR::Bxb1int-attB ΔampC::*P_{PP2688}*:T7pol*	this work
JE1629	*P. putida KT2440 ΔhsdR::Bxb1int-attB ΔampC::*P_{urtA}*:T7pol*	this work
JE1633	*P. putida KT2440 ΔhsdR::Bxb1int-attB ΔampC::*P_{glnK}*:T7pol*	this work
JE1651	*P. putida KT2440 ΔhsdR::Bxb1int-attL:nptII:*P_{T7}*:mNeonGreen:attR ΔampC::*P_{PP2685}*:T7pol*	this work
JE1652	*P. putida KT2440 ΔhsdR::Bxb1int-attL:nptII:*P_{T7}*:mNeonGreen:attR ΔampC::*P_{PP2688}*:T7pol*	this work
JE1653	*P. putida KT2440 ΔhsdR::Bxb1int-attL:nptII:*P_{T7}*:mNeonGreen:attR ΔampC::*P_{urtA}*:T7pol*	this work
JE1654	*P. putida KT2440 ΔhsdR::Bxb1int-attL:nptII:*P_{T7}*:mNeonGreen:attR ΔampC::*P_{glnK}*:T7pol*	this work
JE1655	*P. putida KT2440 ΔhsdR::Bxb1int-attL:nptII:mNeonGreen*(promoterless)*:attR*	this work
JE1657	*P. putida KT2440 ΔhsdR::Bxb1int-attL:nptII:*P_{T7}*:mNeonGreen:attR*	this work
JE2113	*P. putida KT2440 ΔhsdR::Bxb1int-attB ΔampC::lysY:*P_{urtA}*:T7_RNAP*	this work
JE2211	*P. putida KT2440 ΔhsdR::Bxb1int-attL:nptII:*P_{tac}*:mNeonGreen:attR ΔampC::lysY:*P_{urtA}*:T7pol*	this work
JE2212	*P. putida KT2440 ΔhsdR::Bxb1int-attL:nptII:*P_{T7}*:mNeonGreen:attR ΔampC::lysY:*P_{urtA}*:T7pol*	this work
JE3215	*P. putida KT2440 ΔhsdR::Bxb1int-attB ΔampC::lysY:*P_{urtA}*:T7pol Δ*$phaC_1ZC_2$	this work
JE3221	*P. putida KT2440 ΔhsdR::Bxb1int-attL:nptII:PT7:cadA:attR ΔampC::lysY:PurtA:T7_RNAP ΔampC::lysY:*P_{urtA}*:T7pol Δ*$phaC_1ZC_2$	this work
JE3674	*P. putida KT2440 ΔhsdR::Bxb1int-attB ΔampC::lysY:*P_{urtA}*:T7pol Δ*$phaC_1ZC_2$ icd^{GTG}:idh^{GTG}	this work
JE3681	*P. putida KT2440 ΔhsdR::Bxb1int-attB ΔampC::lysY:*P_{urtA}*:T7_RNAP Δ*$phaC_1ZC_2$ icd^{TTG}:idh^{TTG}	this work
JE3713	*P. putida KT2440 ΔhsdR::Bxb1int-attL:nptII:*P_{T7}*:cadA:attR ΔampC::lysY:*P_{urtA}*:T7pol Δ*$phaC_1ZC_2$ icd^{GTG}:idh^{GTG}	this work
JE3715	*P. putida KT2440 ΔhsdR::Bxb1int-attL:nptII:*P_{T7}*:tad1:adi1:attR ΔampC::lysY:*P_{urtA}*:T7_RNAP Δ*$phaC_1ZC_2$ icd^{GTG}:idh^{GTG}	this work
JE3717	*P. putida KT2440 ΔhsdR::Bxb1int-attL:nptII:*P_{T7}*:cadA:attR ΔampC::lysY:*P_{urtA}*:T7pol Δ*$phaC_1ZC_2$ icd^{TTG}:idh^{TTG}	this work
JE3719	*P. putida KT2440 ΔhsdR::Bxb1int-attL:nptII:*P_{T7}*:tad1:adi1:attR ΔampC::lysY:*P_{urtA}*:T7_RNAP Δ*$phaC_1ZC_2$ icd^{TTG}:idh^{TTG}	this work
JE3729	*P. putida KT2440 ΔhsdR::Bxb1int-attL:nptII:*P_{T7}*:mKate2:attR ΔampC::lysY:*P_{urtA}*:T7pol*	this work
JE3730	*P. putida KT2440 ΔhsdR::Bxb1int-attL:nptII:*P_{T7_C4}*:mKate2:attR ΔampC::lysY:*P_{urtA}*:T7pol*	this work
JE3732	*P. putida KT2440 ΔhsdR::Bxb1int-attL:nptII:*P_{T7_H10}*:mKate2:attR ΔampC::lysY:*P_{urtA}*:T7pol*	this work
JE3734	*P. putida KT2440 ΔhsdR::Bxb1int-attL:nptII:*P_{T7_H9}*:mKate2:attR ΔampC::lysY:*P_{urtA}*:T7pol*	this work
JE3736	*P. putida KT2440 ΔhsdR::Bxb1int-attL:nptII:*P_{T7_G6}*:mKate2:attR ΔampC::lysY:*P_{urtA}*:T7pol*	this work
JE3738	*P. putida KT2440 ΔhsdR::Bxb1int-attL:nptII:*P_{tac}*:mKate2:attR ΔampC::lysY:*P_{urtA}*:T7pol*	this work
JE669	*P. putida KT2440 ΔhsdR::Bxb1int-attB Δ*$phaC_1ZC_2$	this work
JE4296	*P. putida KT2440 ΔhsdR::Bxb1int-attB Δ*$phaC_1ZC_2$ icd^{GTG}:idh^{GTG}	this work
JE4274	*P. putida KT2440 ΔhsdR::Bxb1int-attB Δ*$phaC_1ZC_2$ icd^{TTG}:idh^{TTG}	this work

JE4305	*P. putida KT2440 ΔhsdR::Bxb1int-attL:nptII:*P_{tac}*:cadA:attR*	this work
JE4306	*P. putida KT2440 ΔhsdR::Bxb1int-attL:nptII:*P_{tac}*:cadA:attR* $\Delta phaC_1ZC_2$	this work
JE4307	*P. putida KT2440 ΔhsdR::Bxb1int-attL:nptII:*P_{tac}*:cadA:attR* $\Delta phaC_1ZC_2$ $icd^{TTG}:idh^{TTG}$	this work
JE4308	*P. putida KT2440 ΔhsdR::Bxb1int-attL:nptII:*P_{tac}*:cadA:attR* $\Delta phaC_1ZC_2$ $icd^{GTG}:idh^{GTG}$	this work
JE3843	*P. putida KT2440 ΔhsdR::Bxb1int-attB ΔampC::lysY:*P_{urtA}*:T7pol* $\Delta phaC_1ZC_2$ $icd^{TTG}:idh^{TTG}$ $\Delta gcd::araE_1\text{-}araC_2D_2A_2B_2E_2$	this work
JE3887	*P. putida KT2440 ΔhsdR::Bxb1int-attB ΔampC::lysY:*P_{urtA}*:T7polP* $\Delta phaC_1ZC_2$ $icd^{TTG}:idh^{TTG}$ $\Delta gcd::araE_1\text{-}araC_2D_2A_2B_2E_2$ *fpvA:xylE-xylDCBC*	this work
JE3903	*P. putida KT2440 ΔhsdR::Bxb1int- attL:nptII:*P_{T7}*:cadA:attR ΔampC::lysY:*P_{urtA}*:T7pol* $\Delta phaC_1ZC_2$ $icd^{TTG}:idh^{TTG}$ $\Delta gcd::araE_1\text{-}araC_2D_2A_2B_2E_2$ *fpvA:xylE-xylDCBC*	this work
JE3802	*P. putida KT2440 ΔhsdR::Bxb1int- attL:nptII:*P_{tac}*:cadA:attR ΔampC::lysY:*P_{urtA}*:T7pol* $\Delta phaC_1ZC_2$ $icd^{TTG}:idh^{TTG}$	this work
JE90+pJE1045 ATGGTC	*P. putida KT2440 ΔhsdR::Bxb1int-attL:nptII:*P_{tac}*:*$mNeonGreen^{ATGGTC}$*:attR*	this work
JE90+pJE1045 GTGGTC	*P. putida KT2440 ΔhsdR::Bxb1int-attL:nptII:*P_{tac}*:*$mNeonGreen^{GTGGTC}$*:attR*	this work
JE90+pJE1045 TTGGTC	*P. putida KT2440 ΔhsdR::Bxb1int-attL:nptII:*P_{tac}*:*$mNeonGreen^{TTGGTC}$*:attR*	this work
Plasmids		
pJE382	pUC origin, *nptII*, *sacB*, *mcs-lacZa*	this work
pK18mobsacB	pUC origin, *nptII*, *sacB*, P_{lac}*:mcs-lacZa*	[2]
pLysS	p15A origin, *cat*, *lysS*	[3, 4]
pJE990	pUC origin, *nptII*, *mNeonGreen* (promoterless), *Bxb1 attP*	[1]
pJE387	pK18mobsacB *ΔampC*	this work
pJE473	pJE382 $\Delta phaC_1ZC_2$	this work
pJE1031	pJE382 *ΔampC*	this work
pJE1032	pJE382 *ΔampC::*P_{PP2685}*:T7pol*	this work
pJE1033	pJE382 *ΔampC::*P_{PP2688}*:T7pol*	this work
pJE1037	pJE382 *ΔampC::*P_{urtA}*:T7pol*	this work
pJE1039	pJE382 *ΔampC::*P_{glnK}*:T7pol*	this work
pJE1040	pJE990 P_{T7}:*mNeonGreen*	this work
pJE1045	pJE990 P_{tac}:*mNeonGreen*	this work
pJE1118	pJE990 P_{T7_C4}:*mNeonGreen*	this work
pJE1119	pJE990 P_{T7_H10}:*mNeonGreen*	this work
pJE1120	pJE990 P_{T7_H9}:*mNeonGreen*	this work
pJE1121	pJE990 P_{T7_G6}:*mNeonGreen*	this work
pJE1180	pJE382 *ΔampC::lysY:*P_{glnK}*:T7pol*	this work
pJE1380	pJE990 P_{T7}:*cadA*	this work
pJE1390	pJE990 P_{tac}:*cadA*	this work
pJE1443	pJE990 P_{T7}:*tad1:adi1*	this work
pJE1444	pJE382 $icd^{GTG}:idh^{GTG}$	this work
pJE1445	pJE382 $icd^{TTG}:idh^{TTG}$	this work
pJE1454	pJE990 P_{T7}:*mKate2*	this work
pJE1455	pJE990 P_{T7_C4}:*mKate2*	this work

pJE1456	pJE990 P_{T7_H10}:*mKate2*	this work
pJE1457	pJE990 P_{T7_H9}:*mKate2*	this work
pJE1458	pJE990 P_{T7_G6}:*mKate2*	this work
pGW55	pJE990 P_{tac}:*mKate2*	this work
pJE1345	pJE382 Δ*gcd::araE*$_1$-*araC*$_2$*D*$_2$*A*$_2$*B*$_2$*E*$_2$	this work
pJE1479	pJE382 *fpvA:xylE-xylDCBC*	this work
pJE365	pK18mobsacB *Δgcd*	this work
pJE1045-ATGGTC	pJE1045 with second mNeonGreen codon swapped to GTC	this work
pJE1045-GTGGTC	pJE1045 with second mNeonGreen codon swapped to GTC, and start codon swapped to GTG	this work
pJE1045-TTGGTC	pJE1045 with second mNeonGreen codon swapped to GTC, and start codon swapped to TTG	this work

Supplementary Table 6. DNA oligos used in this work.

Oligo name	Oligo sequence (5'-3')	Purpose
oJE255	attaatgcagctggcacgac	primers for screening insertions into the MCS of pJE382
oJE256	agctagcttatcgccattcg	primers for screening insertions into the MCS of pJE382
oJE331	tagctcactcaggaaacagctatgacatgattacgaattcGACCGAAAACATCGGTGC	amplification of homology arms to construction pJE473 for deletion of phaC1ZC2
oJE332	tcagcacgtaggtgcctTCTAGAgtctattgtaGGATCCTCTACGACGCTCCGTTG	amplification of homology arms to construction pJE473 for deletion of phaC1ZC2
oJE333	aCAACGGAGCGTCGTAGAGGATCCtacaatagacTCTAGAAGGCACCTACGT GCTG	amplification of homology arms to construction pJE473 for deletion of phaC1ZC2
oJE334	ccagtcacgacgttgtaaaacgacggccagtgccaagcttGCAGCCAAAACCGCAG	amplification of homology arms to construction pJE473 for deletion of phaC1ZC2
oJE335	cagtaccaggcattgctgaa	screening deletion of phaC1ZC2 (flanking)
oJE336	gccaaggcagcagctaag	screening deletion of phaC1ZC2 (flanking)
oJE337	TGGAGCTGAAGAACGTGTTG	screening deletion of phaC1ZC2 (internal to phaC1)
oJE338	CTCGTCGACAAACAAAGCAA	screening deletion of phaC1ZC2 (internal to phaC1)
oJE92	ccagtcacgacgttgtaaaacgacggccagtgccaagcttGTAACCACGGCCTCACTGA A	amplification of ampC deletion homology arms for construction of pJE1031
oJE608	tagctcactcaggaaacagctatgacatgattacgaattcCTTGCCTCTGCCGGAAAC	amplification of ampC deletion homology arms for construction of pJE1031
oJE609	CTGTCGTTTTGTCCGACAATCAACGCGAGCGttaggatccCATCGCCAGTGAC AGACTG	Amplifies PP_2685 promoter with overlaps to construct pJE1032
oJE610	TGTCAGAGAAGTCGTTCTTAGCGATGTTAATCGTGTTCATGCGGTTTCCCTT GTGTTG	Amplifies PP_2685 promoter with overlaps to construct pJE1032
oJE611	CTGTCGTTTTGTCCGACAATCAACGCGAGCGttaggatccGCCCGGGTCAAAA GCG	Amplifies PP_2688 promoter with overlaps to construct pJE1033
oJE612	TGTCAGAGAAGTCGTTCTTAGCGATGTTAATCGTGTTCATACCCACTCCTTG CCGCCGTT	Amplifies PP_2688 promoter with overlaps to construct pJE1033
oJE619	CTGTCGTTTTGTCCGACAATCAACGCGAGCGttaggatccATGGCCTCGGGGG CTGT	Amplifies PP_4841 promoter with overlaps to construct pJE1037
oJE620	TGTCAGAGAAGTCGTTCTTAGCGATGTTAATCGTGTTCATGTGCTCTCTCCG CTGAGT	Amplifies PP_4841 promoter with overlaps to construct pJE1037
oJE623	CTGTCGTTTTGTCCGACAATCAACGCGAGCGttaggatccGCTGCGCACCGAA ATTGTG	Amplifies PP_5234 promoter with overlaps to construct pJE1039
oJE624	TGTCAGAGAAGTCGTTCTTAGCGATGTTAATCGTGTTCATGAAACTCTCTCC CGATTTGG	Amplifies PP_5234 promoter with overlaps to construct pJE1039
oJE625	ATGAACACGATTAACATCGCTAAG	amplification of T7 RNAP for construction of pJE1032, 1033, 1037, 1039
oJE626	GTAAAAATTGCCATCCCAACAGC	amplification of T7 RNAP for construction of pJE1032, 1033, 1037, 1039
oJE627	GAGCATCAATATGCAATGCTGTTG	amplification of double terminator from Dbl_term_T7 gBlock for construction of pJE1032, 1033, 1037, 1039
oJE628	CGCTCAACGGACACGCT	amplification of double terminator from Dbl_term_T7 gBlock for construction of pJE1032, 1033, 1037, 1039
oJE629	GACCATTACGGTGAGCGTTT	Amplifies an internal fragment of T7_RNAP for PCR screening
oJE630	CGGGTTGAACATTGACACAG	Amplifies an internal fragment of T7_RNAP
oJE631	CTCAACAAGCGCGTAGG	Internal sequencing primer for T7 RNAP gene
oJE632	GTTCATGCTTGAGCAAGCC	Internal sequencing primer for T7 RNAP gene
oJE633	GGTGTTACTCGCAGTGTGAC	Internal sequencing primer for T7 RNAP gene
oJE634	gtctTAATACGACTCACTATAGGGAGAGACCTGGAATTGTGAGCGGATAAC AATT	Anneal with oJE634 to construct T7 promoter for cloning of pJE1040
oJE635	taagAATTGTTATCCGCTCACAATTCCAGGTCTCTCCCTATAGTGAGTCGTAT TA	Anneal with oJE635 to construct T7 promoter for cloning of pJE1040
oJE535	GTTgctagcGTCGGGGTTTGTA	For screening of genomic integration of pJE990/991 and its derivatives into JE90 derivative strains, as well as plasmid sequencing

oJE536	aaaaccgcccagtctagctatcg	For screening of genomic integration of pJE990/991 and its derivatives into JE90 derivative strains, as well as plasmid sequencing
oJE93	GGCGTTGCTGGAAGAGTATT	flanking primers for screening ampC deletion
oJE94	ACCACTGCCAGCAGAATTG	flanking primers for screening ampC deletion
oJE546	gctgttgccatcgatcagt	amplifies internal 851 bp fragment of ampC. Used for screening deletion.
oJE547	acgaccagttacaggccaag	amplifies internal 851 bp fragment of ampC. Used for screening deletion.
oJE177	GGGAGACGGCTTCATCATG	Amplififes sequence inserted between homology arms of pJE387/1031
oJE178	ATCACTGTATCCATCTTGTCATG	Amplififes sequence inserted between homology arms of pJE387/1031
oJE826	gtctTAATACGACTCACTAtcaaggaaGACCTGGAATTGTGAGCGGATAACAATT	cloning T7_C4 promoter into pJE990
oJE827	taagAATTGTTATCCGCTCACAATTCCAGGTCttccttgaTAGTGAGTCGTATTA	cloning T7_C4 promoter into pJE990
oJE828	gtctTAATACGACTCACTAcggaagaaGACCTGGAATTGTGAGCGGATAACAATT	cloning T7_H10 promoter into pJE990
oJE829	taagAATTGTTATCCGCTCACAATTCCAGGTCttcttccgTAGTGAGTCGTATTA	cloning T7_H10 promoter into pJE990
oJE830	gtctTAATACGACTCACTAatactgaaGACCTGGAATTGTGAGCGGATAACAATT	cloning T7_H9 promoter into pJE990
oJE831	taagAATTGTTATCCGCTCACAATTCCAGGTCttcagtatTAGTGAGTCGTATTA	cloning T7_H9 promoter into pJE990
oJE832	gtctTAATACGACTCACTAtttcggaaGACCTGGAATTGTGAGCGGATAACAATT	cloning T7_G6 promoter into pJE990
oJE833	taagAATTGTTATCCGCTCACAATTCCAGGTCttccgaaaTAGTGAGTCGTATTA	cloning T7_G6 promoter into pJE990
oJE817	cccgaaagggggccttttttcgttttggtccactagtCACTATCGACTACGCGATCATG	amplify part of pLysS for construction of pJE1110
oJE818	GAAGGCGCTGGTCTTCGCGCCCATCATGAGGTGGCGCCGTACGCTTGCCCTTCGTTCGAC	amplify part of pLysS for construction of pJE1110
oJE819	TCTCCCACCAACGCTTAAGGTCGAACGAAGGGCAAGCGTACGGCGCCACCTCATGAT	amplify part of pLysS for construction of pJE1110
oJE820	CAGGTCTCTCCCTATAGTGAGTCGTATTAagactactagtCCTGTTGATACCGGGAAGC	amplify part of pLysS for construction of pJE1110
oJE821	TCACGGACACCAACATTCTGAC	sequencing of LysY fragment of pJE1180
oJE1408	GATAACAATTcttaagattaactcacacaggagatatcat	amplification of cadA gBlocks for pJE1380 construction
oJE1409	CCTTGGTAAACATTTTCAGAAAACC	amplification of cadA gBlocks for pJE1380 construction
oJE1410	GAACGCAGCTATGGGGGTTTTCTG	amplification of cadA gBlocks for pJE1380 construction
oJE1411	AAGGCCCCCCGTTAGGGAGGCCTTATTGTTCGTCtctagaTTAGACCAAGG	amplification of cadA gBlocks for pJE1380 construction
oJE1412	TGCATAGCGCAAGCATTGTG	sequencing of cadA in pJE1380
oJE1547	ATTCTAGGCACTGCTGTACTGATAGGGTATTCACGCCGACGATGGAC	amplification of tad1 gBlock for assembly of pJE1443
oJE1548	CGTGTGTTGAGCCGTCCATCGTCGGCGTGAATACCCTATCAGTACAGCAGTG	amplfication of adi1 gBlock for assembly of pJE1443
oJE1554	TGGAATTGTGAGCGGATAACAATTcttaagGTagaTaAGAGCGGGTCATCG	amplfication of tad1 gBlock for assembly of pJE1443
oJE1555	GTTAGGGAGGCCTTATTGTTCGTCtctagaTCAGGACAAGCTCCGGTC	amplfication of adi1 gBlock for assembly of pJE1443
oJE1559	AGCAACGGTTGGATAGCATC	sequencing of tad1
oJE1560	CAGGTCTTTCCCGATGCAAT	sequencing of tad1/downstream genes
oJE1561	AACCGCATCCGTCCGATAC	sequencing of adi1
oJE1564	cactcaggaaacagctatgacatgattacgaattcgccgccatcaagcagtt	amplification of UP homology arm for pJE1444/1445 construction
oJE1565	ggataccagaaaatcaaggttccga	amplification of UP homology arm for pJE1444/1445 construction
oJE1566	tcggaaccttgattttctggtatccCACCGAAgcactactccgctgtcg	amplification of icd/idh promoter region with GTG start codons for pJE1444 construction
oJE1567	tatagatgatcttggaacgggtgggCACgTTTgttaactctctgtgtgctgagc	amplification of icd/idh promoter region with GTG start codons for pJE1444 construction
oJE1568	cccacccgttccaagatcat	amplification of DN homology arm for pJE1444/1445 construction

oJE1569	cacgacgttgtaaaacgacggccagtgccaagcttaacatgatcgggtcgga	amplification of DN homology arm for pJE1444/1445 construction
oJE1570	tcggaaccttgattttctggtatccCAACGAAgcactactccgctgtcg	amplification of icd/idh promoter region with TTG start codons for pJE1445 construction
oJE1571	tatagatgatcttggaacgggtgggCAAgTTTgttaactctctgtgtgctgagc	amplification of icd/idh promoter region with TTG start codons for pJE1445 construction
oJE1572	cgataccacataatcacgcac	sequencing of pJE1444/1445
oJE1573	ctctcgactttccgctcat	sequencing of pJE1444/1445
oJE1574	ttttctggtatccCACCGAA	screening for GTG start codon swap for icd/idh in P. putida
oJE1575	gggtgggCACgTTT	screening for GTG start codon swap for icd/idh in P. putida
oJE1576	gattttctggtatccCAACGAA	screening for TTG start codon swap for icd/idh in P. putida
oJE1577	cgggtgggCAAgTTT	screening for TTG start codon swap for icd/idh in P. putida
oJE1578	gattttctggtatcccatgctt	screening for wild-type start codon for icd/idh in P. putida
oJE1579	gtgggcatgcgg	screening for wild-type start codon for icd/idh in P. putida
oJE1580	gtggcgatcacgtcgtact	screening to ensure that plasmid backbone is removed following start codon swap
oJE1581	aggttggtgatgcctttgtc	screening to ensure that plasmid backbone is removed following start codon swap
oJE1582	aggaatgatcggatggtcag	sequencing of icd promoter region. Use with oJE1581 to amplify region for sequencing.
oJE66	catgtagttgtaggcgtcttc	screening integration of pJE990-derivative plasmids via the Bbx1-phage integrase system
oJE89	acaatttcacacaggaaacagctatgacatgattacgaattcCTTGCCTCTGCCGGAAAC	amplification of ΔampC homology arm for cloning of pJE387
oJE90	AAGGACTCACCtctagaATGGTAcccgggAAGCAAggatcctaaCGCTCGCGTTGATTGT	amplification of ΔampC homology arm for cloning of pJE387
oJE91	GCGAGCGttaggatccTTGCTTcccgggTACCATtctagaGGTGAGTCCTTTTGTGGAG	amplification of ΔampC homology arm for cloning of pJE387
oJE92	ccagtcacgacgttgtaaaacgacggccagtgccaagcttGTAACCACGGCCTCACTGAA	amplification of ΔampC homology arm for cloning of pJE387
oJE69	caattaatgtgagttagctcactcaggaaacagctatgacatga	Quickchange PCR mutagensis primers for deletion of lac promoter from pK18mobsacB
oJE70	tcatgtcatagctgtttcctgagtgagctaactcacattaattg	Quickchange PCR mutagensis primers for deletion of lac promoter from pK18mobsacB
oJE71	gtttcccgactggaaagc	screen for deletion of lac promoter in pK18mobsacB
oJE72	ggtcagcaccgtttctg	screen for deletion of lac promoter in pK18mobsacB
pJE1045-ATGGTCf	TTAACTCACACAGGAGATATCATATGGTCTCCAAAGGGGAAGAGGACAATATG	Mutation of pJE1045 by PCR mutagenesis to construct pJE1045-ATGGTC
pJE1045-ATGGTCr	CATATTGTCCTCTTCCCCTTTGGAGACCATATGATATCTCCTGTGTGAGTTAA	Mutation of pJE1045 by PCR mutagenesis to construct pJE1045-ATGGTC
pJE1045-GTGGTCf	TTAACTCACACAGGAGATATCATGTGGTCTCCAAAGGGGAAGAGGACAATATG	Mutation of pJE1045 by PCR mutagenesis to construct pJE1045-GTGGTC
pJE1045-GTGGTCr	CATATTGTCCTCTTCCCCTTTGGAGACCACATGATATCTCCTGTGTGAGTTAA	Mutation of pJE1045 by PCR mutagenesis to construct pJE1045-GTGGTC
pJE1045-TTGGTCf	TTAACTCACACAGGAGATATCATTTGGTCTCCAAAGGGGAAGAGGACAATATG	Mutation of pJE1045 by PCR mutagenesis to construct pJE1045-TTGGTC
pJE1045-TTGGTCr	CATATTGTCCTCTTCCCCTTTGGAGACCACATGATATCTCCTGTGTGAGTTAA	Mutation of pJE1045 by PCR mutagenesis to construct pJE1045-TTGGTC

Supplementary references

1. Elmore, J.R., Furches, A., Wolff, G.N., Gorday, K. & Guss, A.M. Development of a high efficiency integration system and promoter library for rapid modification of *Pseudomonas putida* KT2440. *Metab Eng Commun* 5, 1-8 (2017).
2. Schafer, A. et al. Small mobilizable multi-purpose cloning vectors derived from the Escherichia coli plasmids pK18 and pK19: selection of defined deletions in the chromosome of Corynebacterium glutamicum. *Gene* 145, 69-73 (1994).
3. Studier, F.W. & Moffatt, B.A. Use of bacteriophage-T7 RNA-polymerase to direct selective high-level expression of cloned genes. *Journal of Molecular Biology* 189, 113-130 (1986).
4. Davanloo, P., Rosenberg, A.H., Dunn, J.J. & Studier, F.W. Cloning and expression of the gene for bacteriophage-T7 RNA-polymerase. *P Natl Acad Sci-Biol* 81, 2035-2039 (1984).
