# Supplementary material for: Production of itaconic acid from alkali pretreated lignin by dynamic two stage bioconversion
Source: Nat Commun. 2021 Apr 15;12:2261. doi: 10.1038/s41467-021-22556-8 (PMC8050072; doi:10.1038/s41467-021-22556-8)
Supplement: Supplementary file 5 — Description of Additional Supplementary Files [file 41467_2021_22556_MOESM5_ESM.pdf]

Description of additional supplementary files

Title: Supplementary Data 1

Description: Genomewide comparative expression data generated by DEseq2.

Title: Supplementary Data 2.

Description: Genomewide comparative expression data generated by DEseq2.

Title: Supplementary Data 3.

Description: Annotated Genbank files for all plasmids used in the study.
